# Supplementary material for: A clade of receptor-like cytoplasmic kinases and 14-3-3 proteins coordinate inositol hexaphosphate accumulation
Source: Nat Commun. 2024 Jun 14;15:5107. doi: 10.1038/s41467-024-49102-6 (PMC11178898; doi:10.1038/s41467-024-49102-6)
Supplement: Supplementary file 1 — Supplementary Information [file 41467_2024_49102_MOESM1_ESM.pdf]

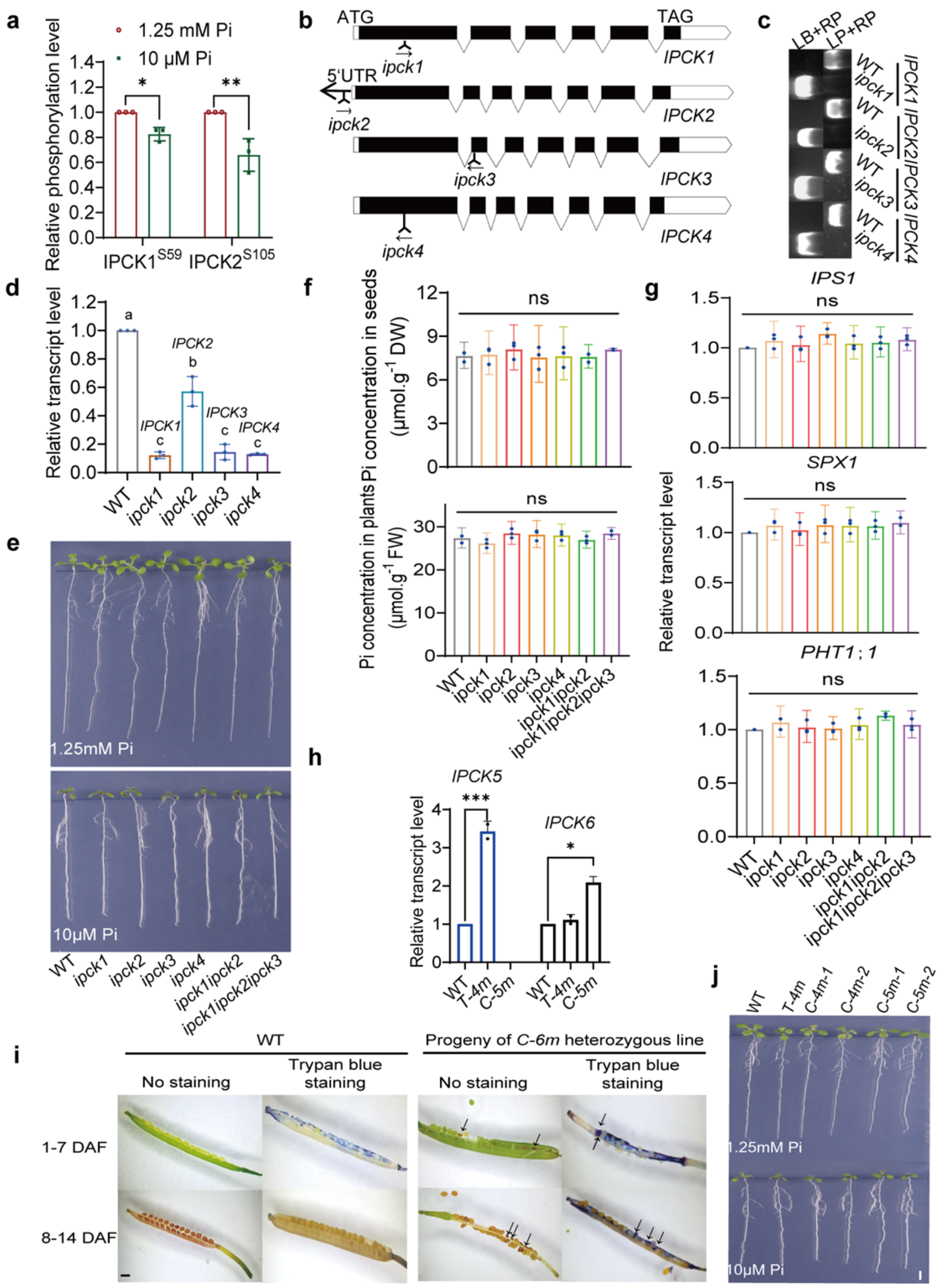

Supplementary Fig. 1 Phenotypical analysis the *ipck* mutants

a Quantitative phosphoproteomics showing the phosphorylation levels of Ser59 in IPCK1 (IPCK1^{S59}) and Ser105 in IPCK2 (IPCK2^{S105}) under 1.25 mM or 10 μM Pi condition. Results are means ± SD from three biological replicates. **b** Diagrams of T-DNA insertion in the indicated mutants. **c** PCR showing the homozygosity of each mutant. **d** Expression analysis of *IPCK1*, *IPCK2*, *IPCK3* and *IPCK4* in the mutants. Values are mean ± SD from three biological replicates. **e**,**j** Morphological observation of mutants grown on 1.25 mM and 10 μM Pi medium for 10 days. Bar = 1 cm. **f** Pi concentration in dry seeds or seedlings grown on 1.25 mM medium for 10 days. Values are mean ± SD from four biological replicates. FW, fresh weight; DW, dry weight. **g** Transcriptional analysis of PSI genes in 10-day-old seedlings grown on 1.25

mM Pi medium. Values are mean ± SD from three biological replicates. **h** Transcriptional analysis of *IPCK5* and *IPCK6* in WT, *T-4m* and *C-5m*. Values are mean ± SD from three biological replicates. **i** The *C-6m* heterozygous plants (*IPCK6* +/- in *C-5m*) showing partial embryonic mortality. Cell death was detected by trypan blue staining. Arrows indicate the positions of some of the aborted seeds, DAF: days after flowering; Bar = 200 μm. All data were analyzed by unpaired t-test, (ns indicates non-significant, *$P < 0.05$, **$P < 0.01$, ***$P < 0.001$, different letters indicate significant difference). All experiments were repeated at least three times with similar results.

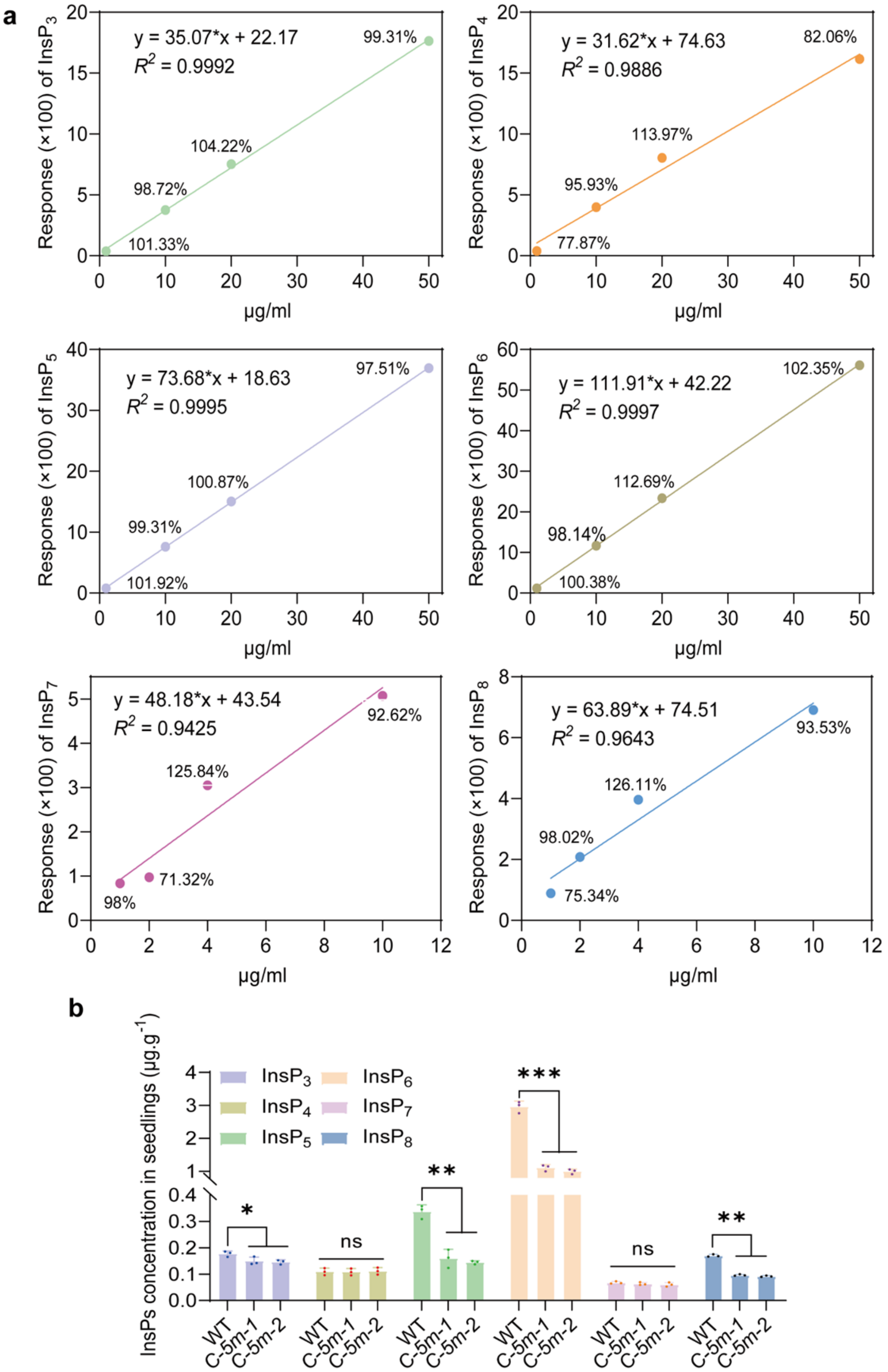

Supplementary Fig. 2 HPLC-MS/MS detection for InsPs

a Calibrations of $InsP_3$ / $InsP_4$ / $InsP_5$ / $InsP_6$ / $InsP_7$ / $InsP_8$. $InsP_3$ / $InsP_4$ / $InsP_5$ / $InsP_6$ with certain concentration gradients (1, 10, 20, 50 μg/ml) and $InsP_7$ (5-$InsP_7$) / $InsP_8$ (1,5-$InsP_8$) with certain concentration gradients (1, 2, 4, 10 μg/ml) were added into 10% ammonia solution, respectively, with a final volume of 100 μl for HPLC-MS/MS

detection. The calibrations curves were fitted based on the concentration gradient and response values. The percentage represents the recovery rate corresponding to each point. **b** The concentration of $InsP_3$ / $InsP_4$ / $InsP_5$ / $InsP_7$ / $InsP_8$ in 10 g of 12-day-old seedlings detected by HPLC-MS/MS. The concentration of $InsP_6$ belongs to the same batch of samples as shown in Fig. 2f. Results are means ± SD from three biological replicates. Data were analyzed by unpaired t-test, (ns indicates non-significant, $^{*}P < 0.05$, $^{**}P < 0.01$, $^{***}P < 0.001$). All experiments were repeated at least three times with similar results.

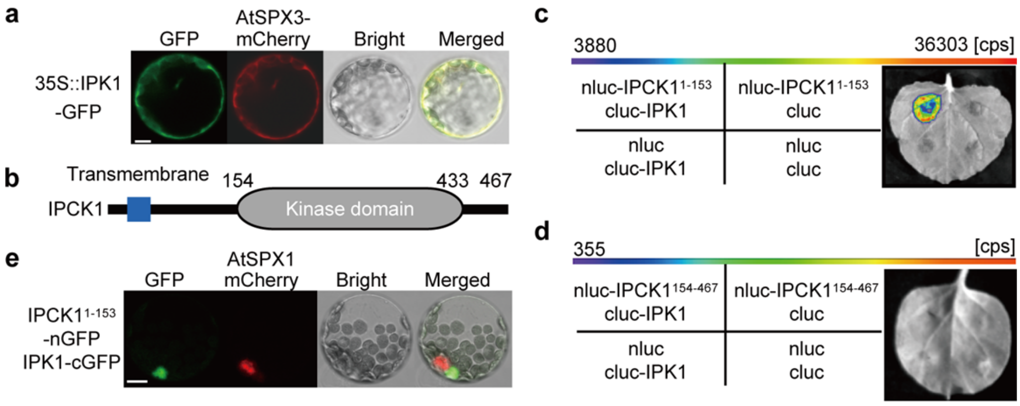

Supplementary Fig. 3 Analysis of the interaction between IPCK1 and IPK1

a Expression and localization of IPK1-GFP fusions in WT mesophyll protoplasts. Bar = 10 μm. **b** The diagram of predicted IPCK1 protein structure. **c,d** Split-LUC assays showing the interaction between truncated IPCK1 and IPK1. Cps means the fluorescence value. **e** BiFC assay showing the interaction between IPCK1$^{1\text{-}153}$ and IPK1 in protoplast. Bar = 10 μm. All experiments were repeated at least three times with similar results.

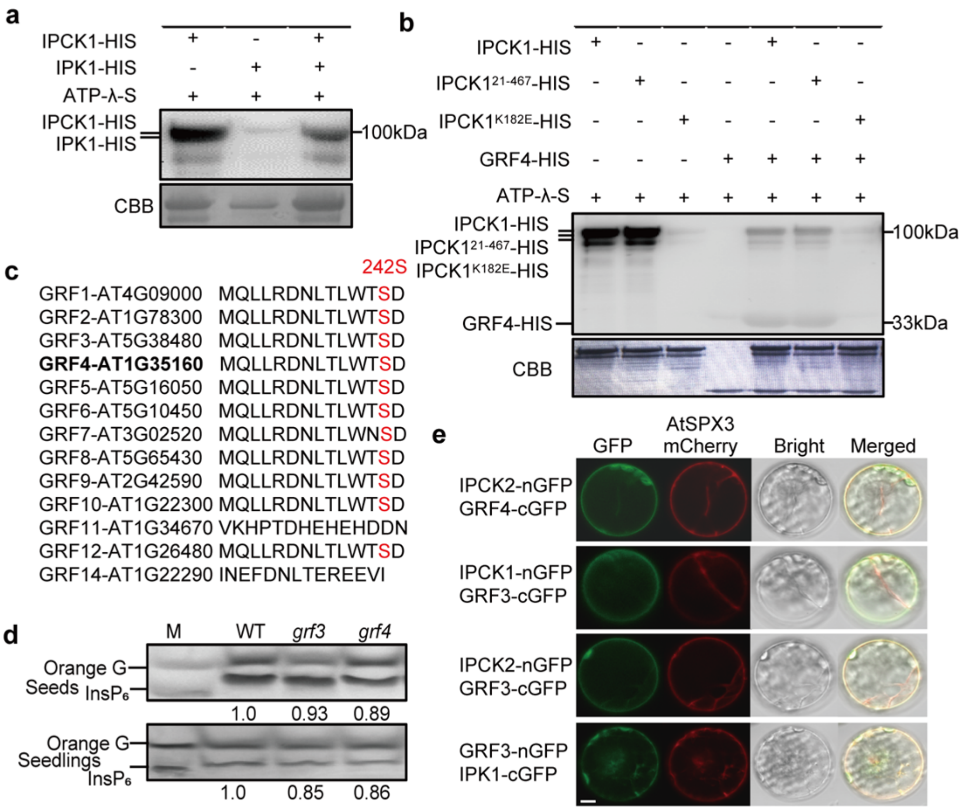

Supplementary Fig. 4 Analysis of GRFs in $InsP_6$ accumulation

a *In vitro* phosphorylation test showing that IPCK1 cannot phosphorylate IPK1. CBB, Coomassie brilliant blue. **b** *In vitro* phosphorylation assay showing that IPCK1 and $IPCK1^{21\text{-}467}$ can phosphorylate GRF4. K182E of IPCK1 is a kinase dead mutation used as a negative control. CBB Coomassie brilliant blue. **c** Protein sequence alignment showing that S242 is highly conserved in GRF family. **d** Isolation and SDS-PAGE analysis of InsPs. **e** BiFC assay showing the interaction between IPCKs and GRF3 in protoplast. Bar = 10 μm. All experiments were repeated at least three times with similar results.

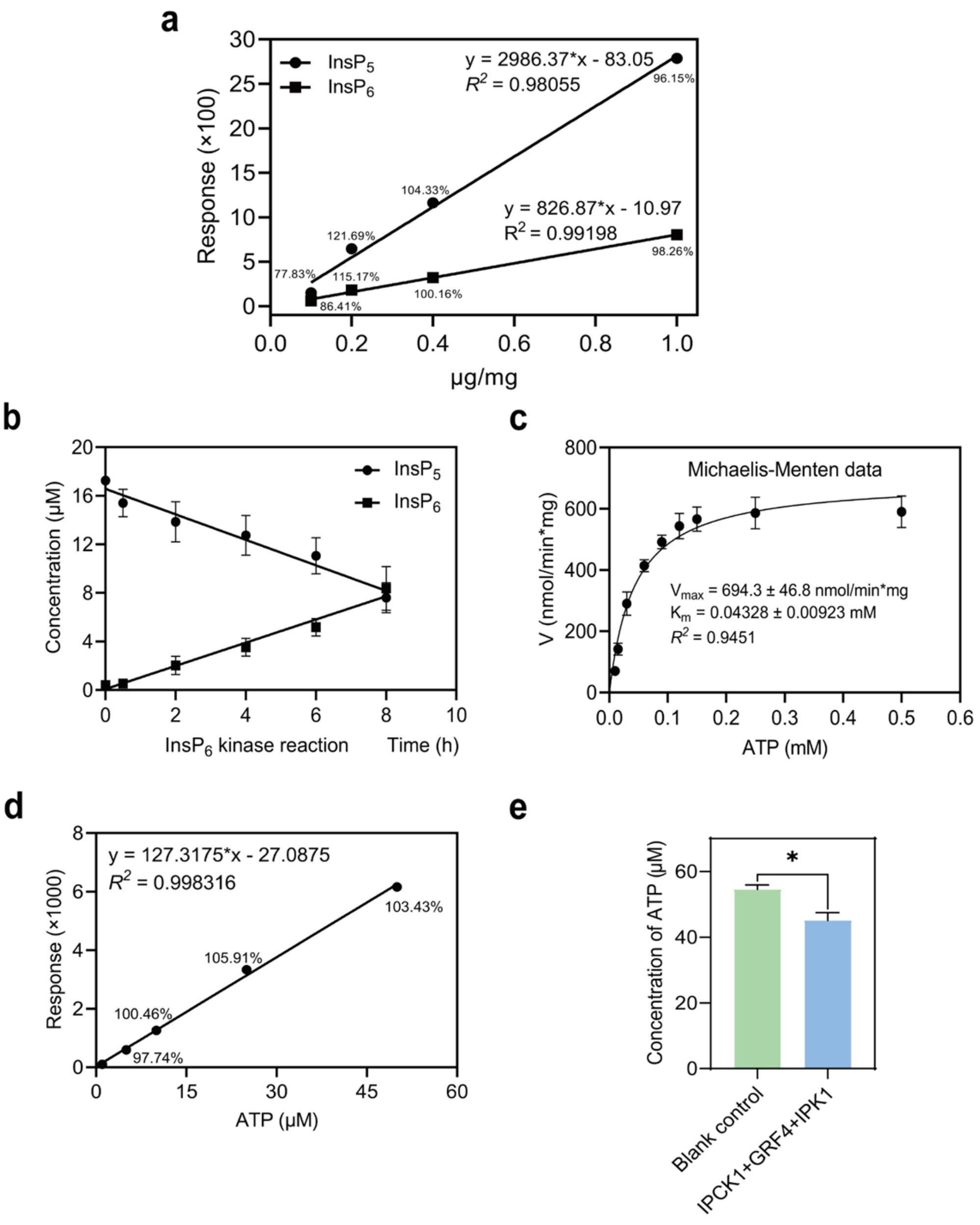

Supplementary Fig. 5 *in vitro* IPK1 activity assay

a Calibrations of $InsP_5$ and $InsP_6$. 0.1, 0.2, 0.4 and 1 μg/mg of $InsP_5$ and $InsP_6$ were added to the reaction buffer (containing 10 mM Tris, pH 8.0, 60 μM ATP) and were detected with HPLC-MS/MS after 6 h of reaction. The percentage represents the recovery rate corresponding to each point. **b** The dynamic concentrations of $InsP_5$ and $InsP_6$ in the IPK1 activity assay. Each concentration was calculated from the integrated HPLC peaks. Values are mean ± SD from three biological replicates. **c** The ATP-dependent kinetic analysis of IPK1 activity. Data were fitted to the Michaelis-Menten equation. Each point is a single measurement from a single experiment. the experiment was repeated three times. K_m and V_{max} (Results are mean ± SD from three biological replicates) are given in the text. Assays were performed in 50 μl of reaction buffers (10 mM Tris, pH 8.0, 39 μM $InsP_5$ and 2.5 μg IPK1-HIS

protein) with corresponding ATP concentration (0.01, 0.015, 0.03, 0.06, 0.09, 0.12, 0.15, 0.25, 0.5 mM). **d** Calibrations of ATP. 1, 5, 10, 25 and 50 μM of ATP were added into 50 μl of reaction buffer (10 mM Tris, pH 8.0, 39 μM $InsP_5$), and were detected with HPLC-MS/MS after 6 h of reaction. The percentage represents the recovery rate corresponding to each point. **e** Detection of the final ATP abundance in the reaction buffer after 6 h of reaction. Blank control presents no protein was added in reaction system. Results are means ± SD from three biological replicates. Data were analyzed by *t*-test (*$P < 0.05$). All experiments were repeated at least three times with similar results.

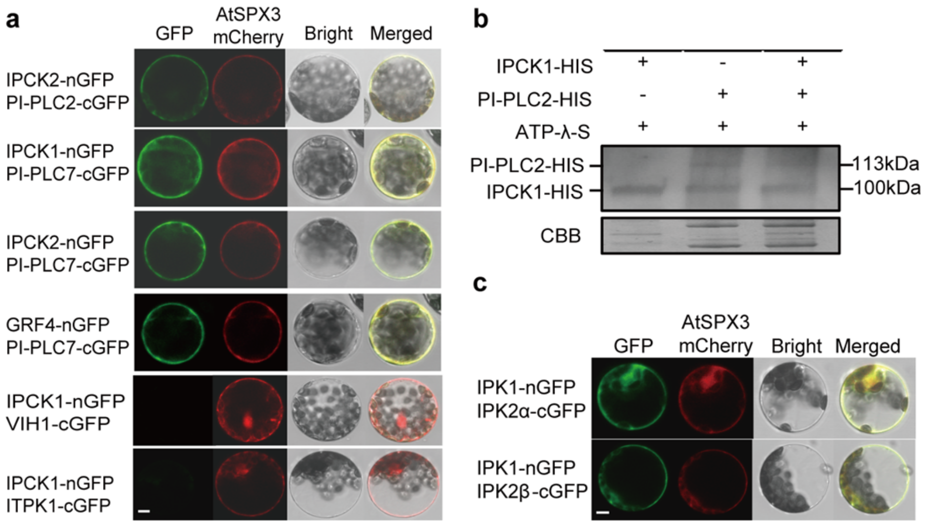

Supplementary Fig. 6 **a** BiFC showing that IPCK1/2 and GRF4 can interact with PI-PLC2/7, and that IPCK1 cannot interact with VIH1/ITPK1 in protoplast. Bar = 10 µm. **b** *In vitro* kinase assay showing that IPCK1 cannot phosphorylate PI-PLC2. CBB Coomassie brilliant blue. **c** BiFC assay showing the interaction between IPK1 and IPK2s in protoplast. Bar = 10 µm. All experiments were repeated at least three times with similar results.

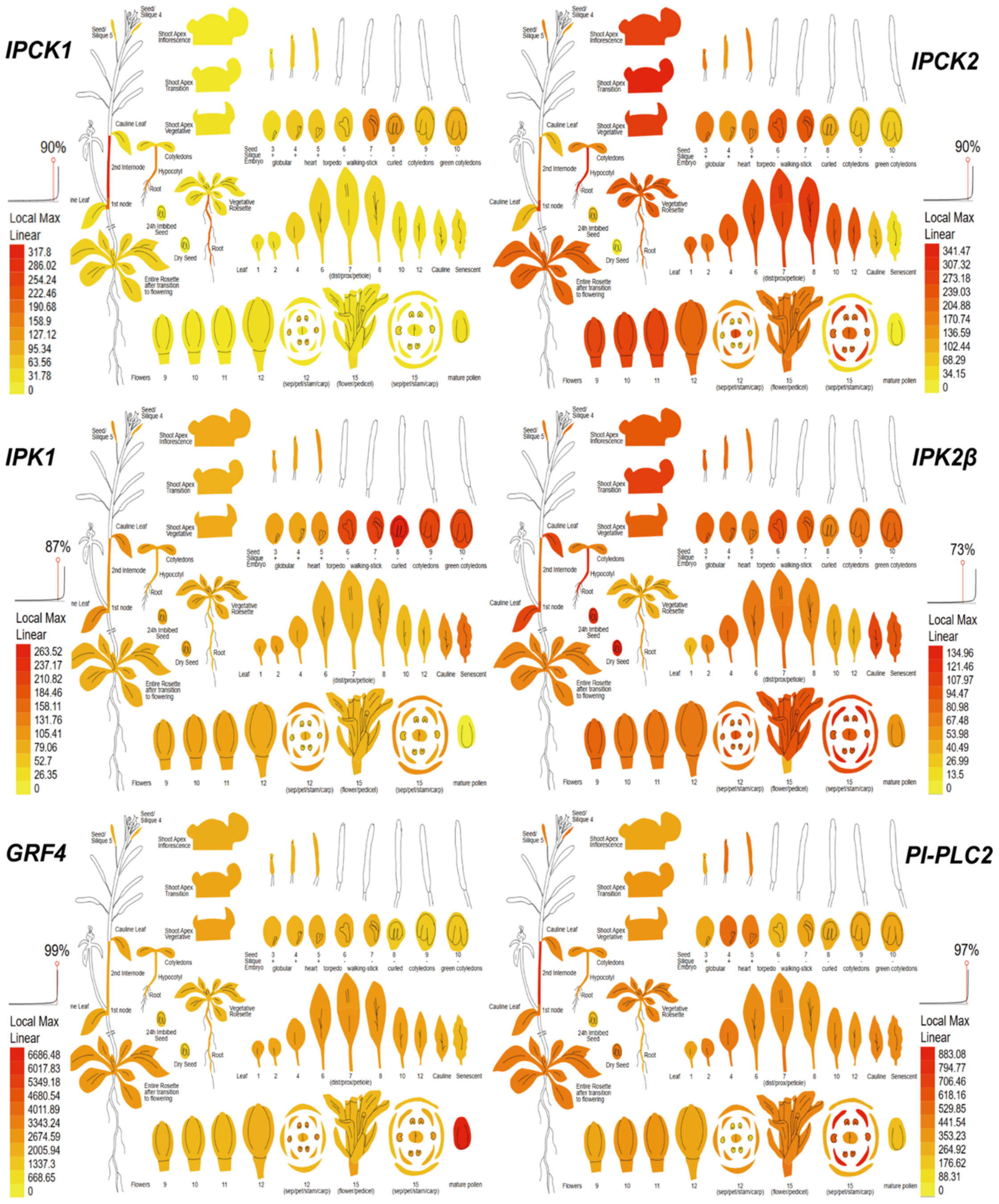

Supplementary Fig. 7 Display of gene expression in different organs from online databases. Transcript abundance of the indicated genes in different Arabidopsis tissues and developmental stages based on Affymetrix ATH1 array data from Schmid et al. (2005), and visualized with the Plant eFP browser (https://bar.utoronto.ca/eplant/; Winter et al. (2007); Waese et al. (2017)).

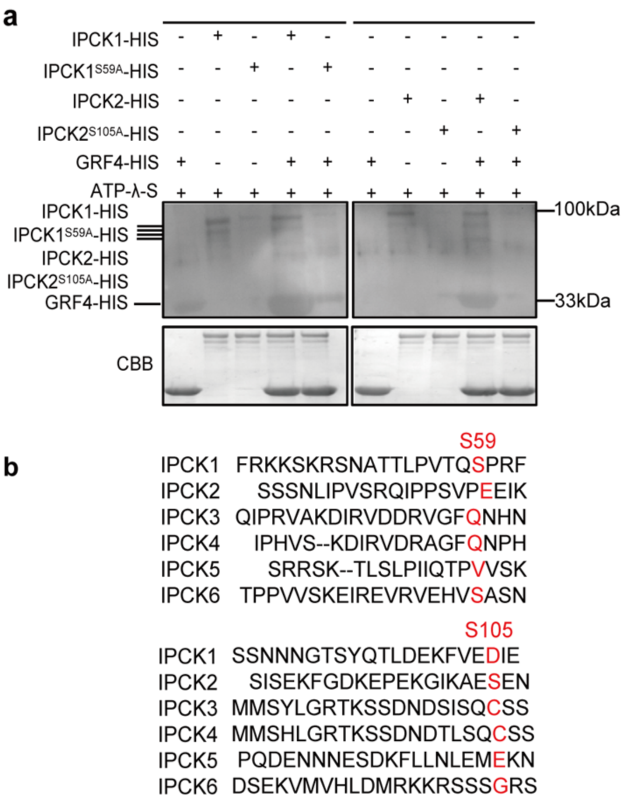

Supplementary Fig. 8 Phosphorylation of IPCK1/2 affects their kinase activity

a *In vitro* kinase assay showing that GRF4 cannot be phosphorylated by IPCK1^{S59A} or IPCK2^{S105A}. CBB Coomassie brilliant blue. All experiments were repeated at least three times with similar results. **b** Protein sequence alignment showing that S59 of IPCK1 and 105S of IPCK2 are not conserved in the RLCK V subfamily.
