# Supplementary material for: A clade of receptor-like cytoplasmic kinases and 14-3-3 proteins coordinate inositol hexaphosphate accumulation
Source: Nat Commun. 2024 Jun 14;15:5107. doi: 10.1038/s41467-024-49102-6 (PMC11178898; doi:10.1038/s41467-024-49102-6)
Supplement: Supplementary file 3 — Description of Additional Supplementary Files [file 41467_2024_49102_MOESM3_ESM.pdf]

Description of Additional Supplementary Files:

Supplementary Data 1: The candidate interacting proteins of IPCK1 from yeast membrane library

Supplementary Data 2: Immunoprecipitation mass spectrometry (IP-MS) analysis results

Supplementary Data 3: Primers used in this study
