# Supplementary material for: A clade of receptor-like cytoplasmic kinases and 14-3-3 proteins coordinate inositol hexaphosphate accumulation
Source: Nat Commun. 2024 Jun 14;15:5107. doi: 10.1038/s41467-024-49102-6 (PMC11178898; doi:10.1038/s41467-024-49102-6)
Supplement: Supplementary file 8 — Source data [file 41467_2024_49102_MOESM8_ESM.zip › Source data/Raw full pictures.pptx]

## Slide 1
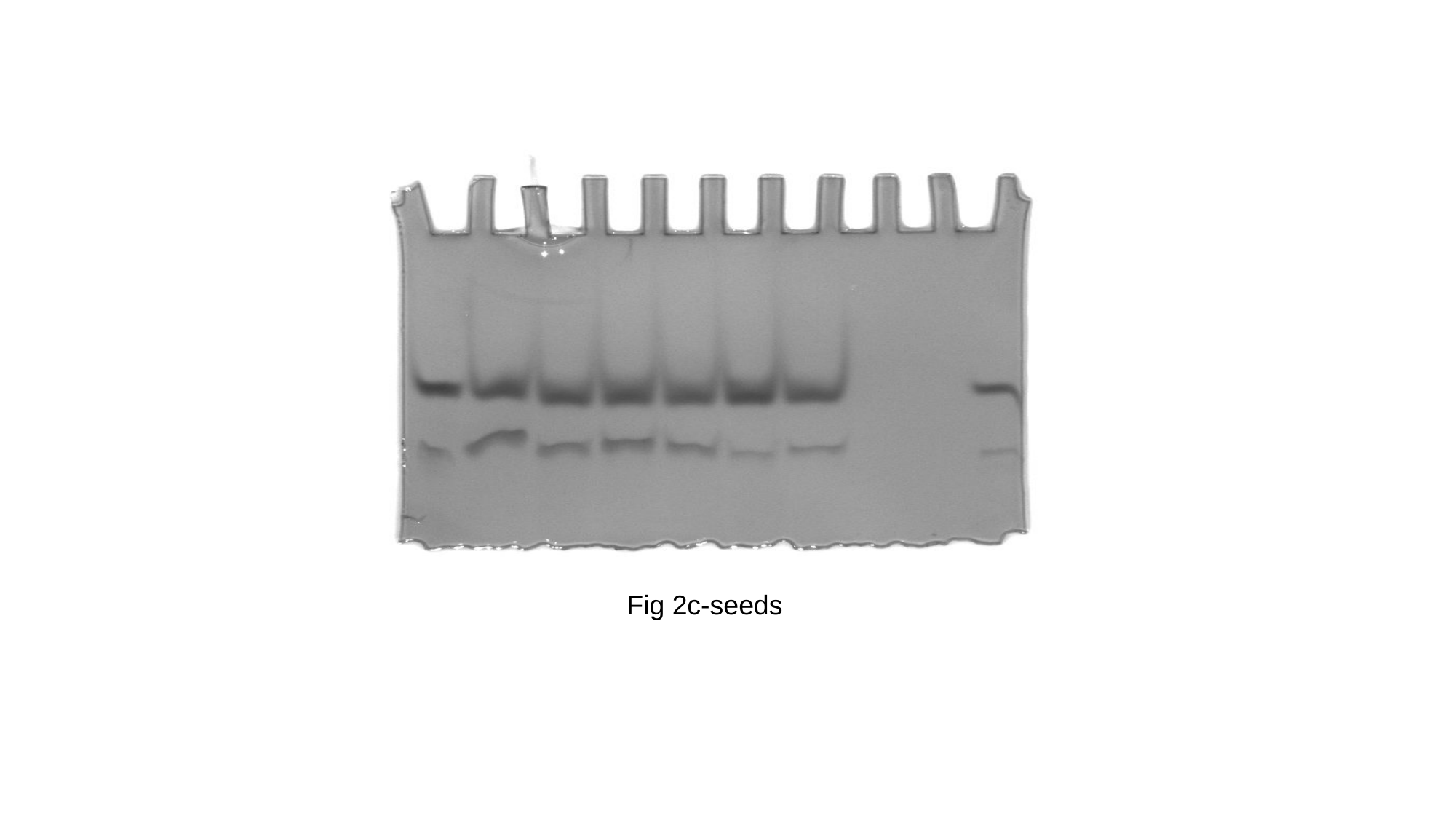

Fig 2c-seeds

## Slide 2
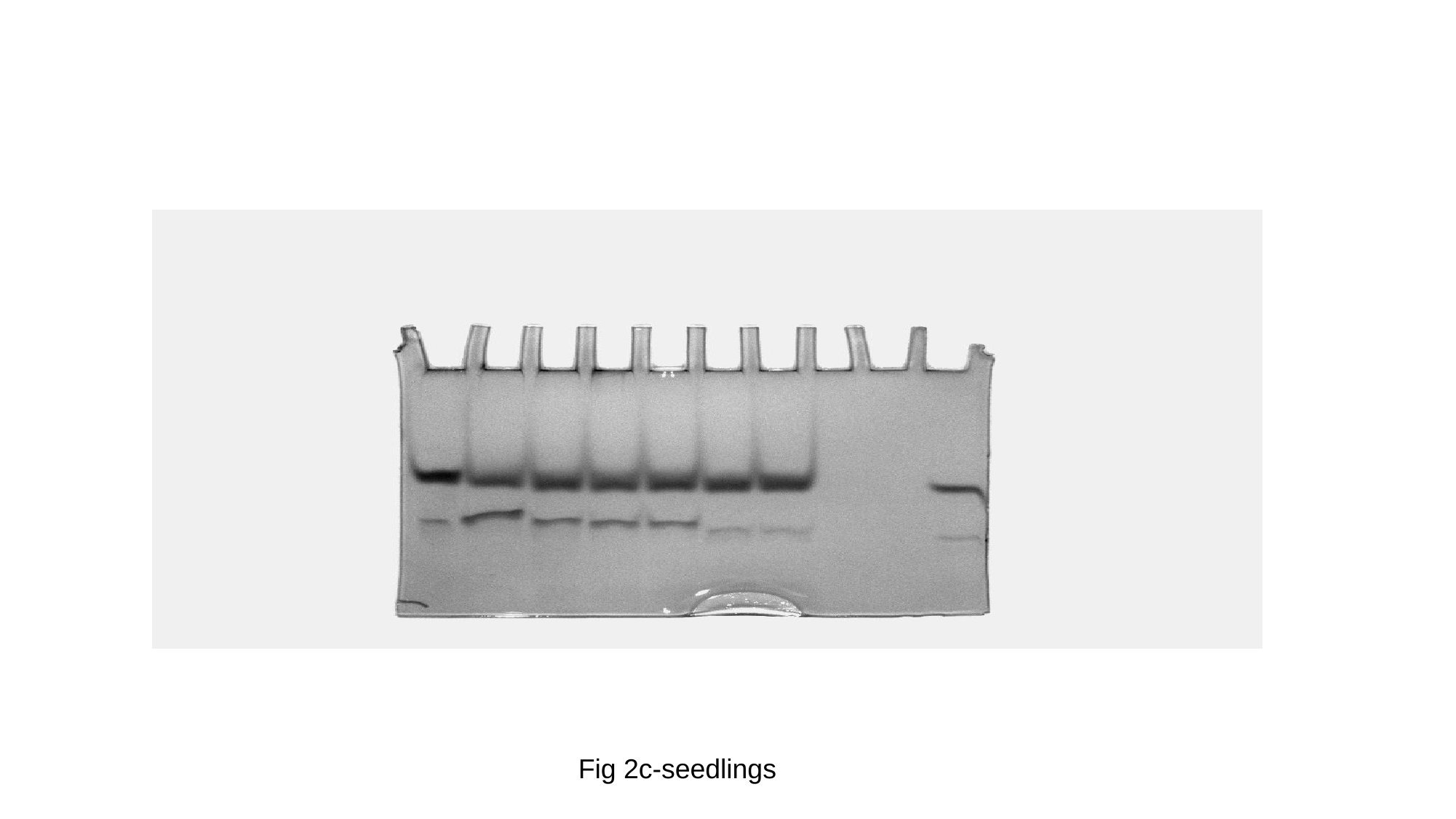

Fig 2c-seedlings

## Slide 3
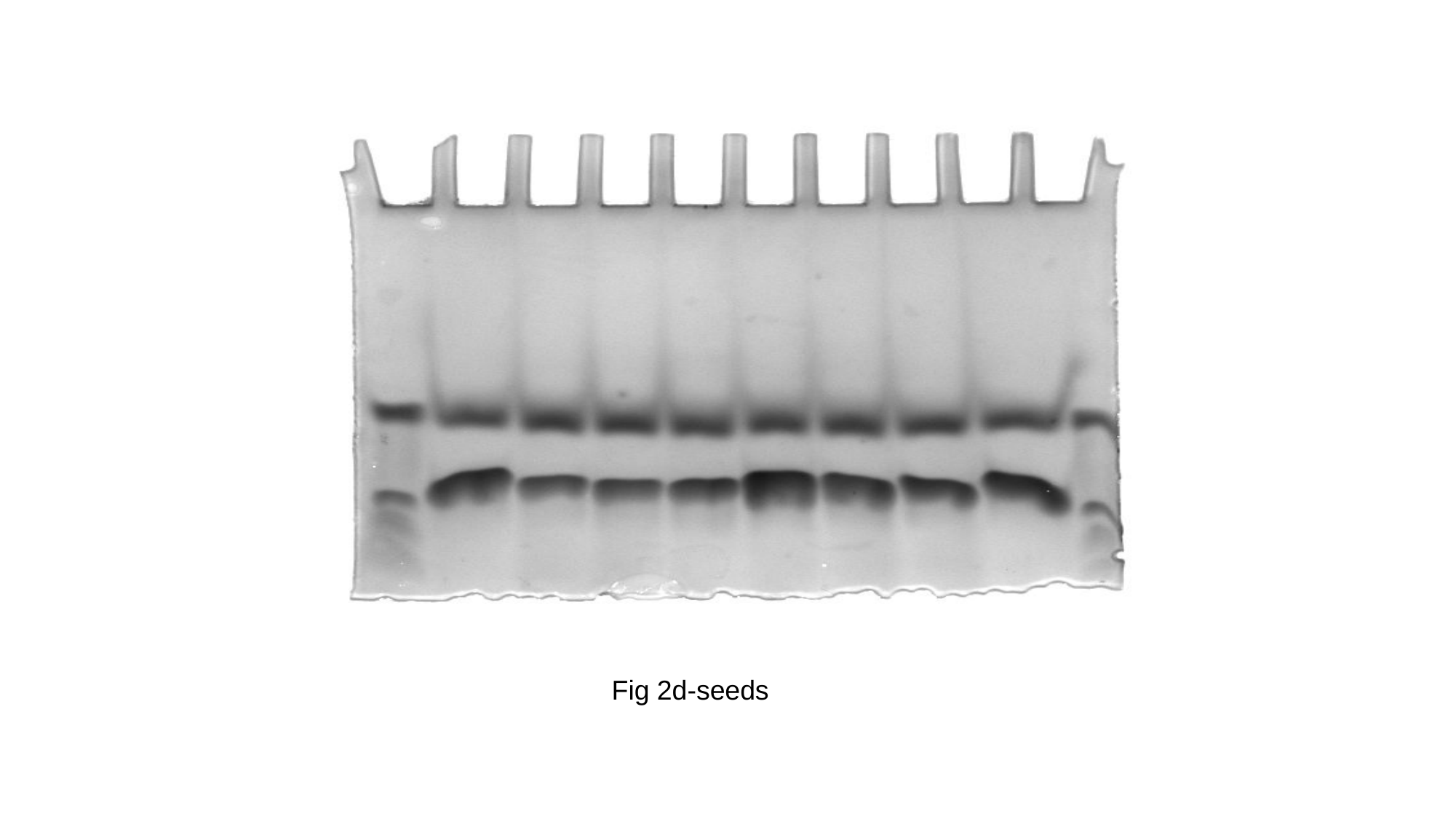

Fig 2d-seeds

## Slide 4
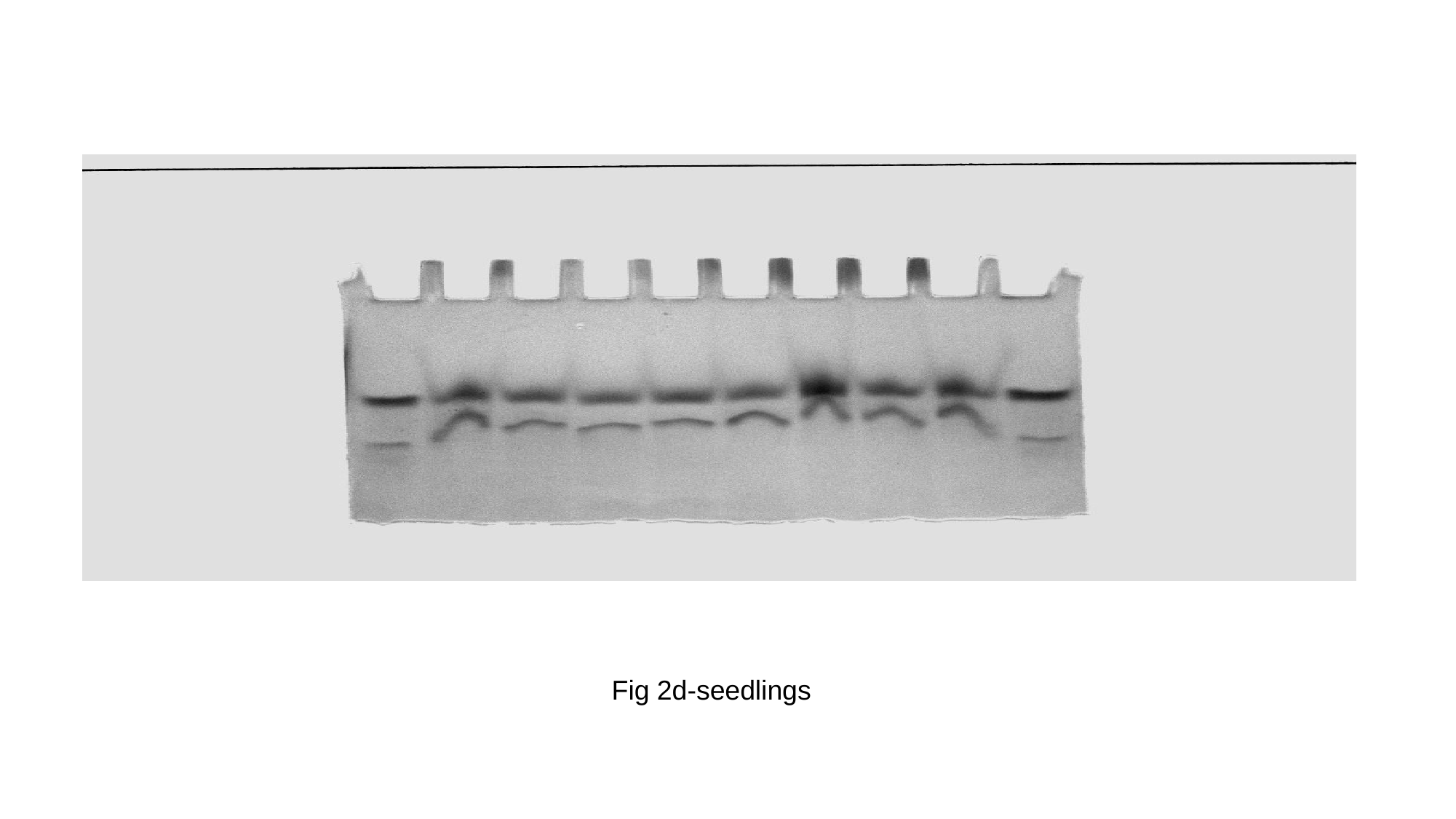

Fig 2d-seedlings

## Slide 5
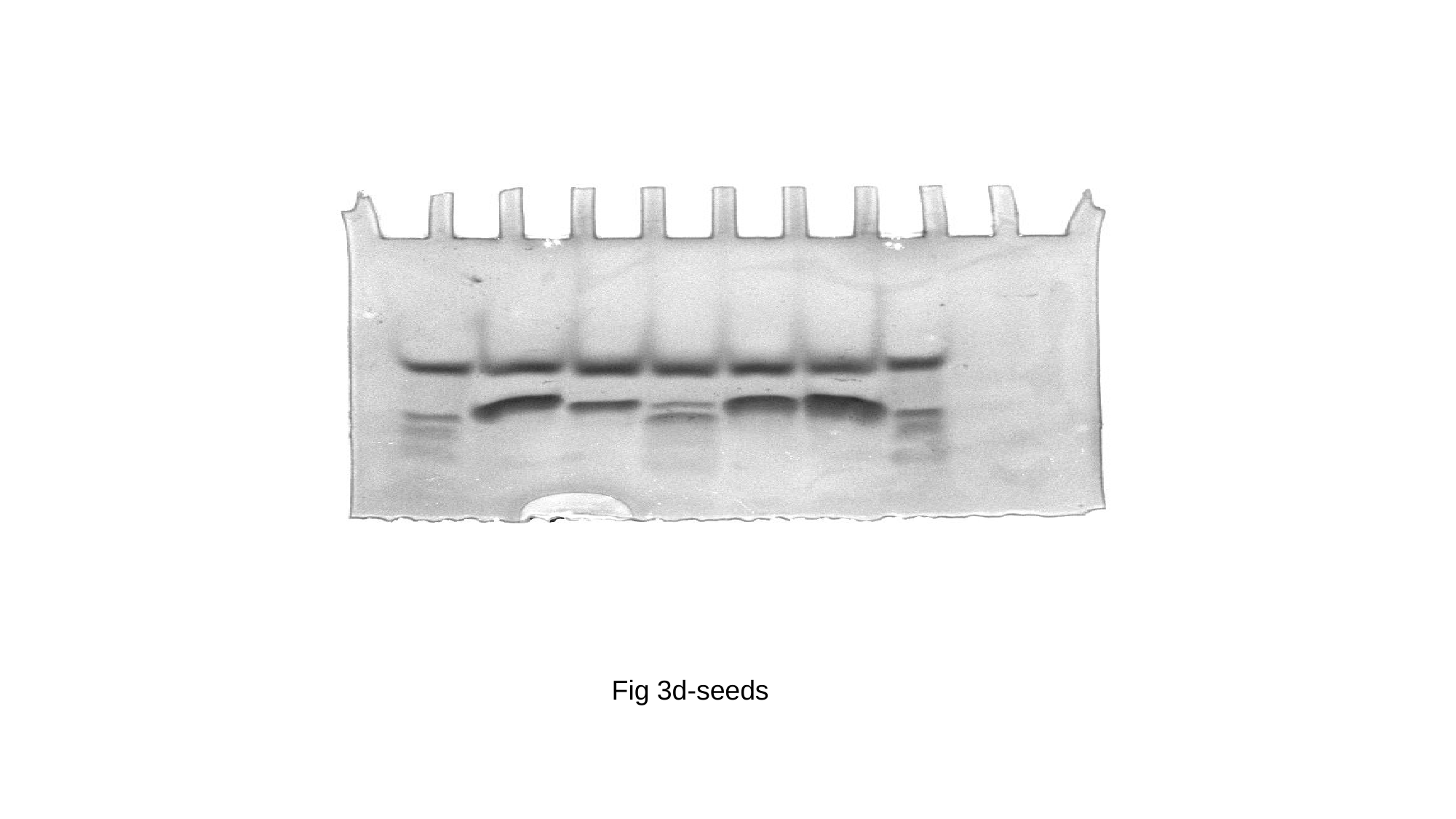

Fig 3d-seeds

## Slide 6
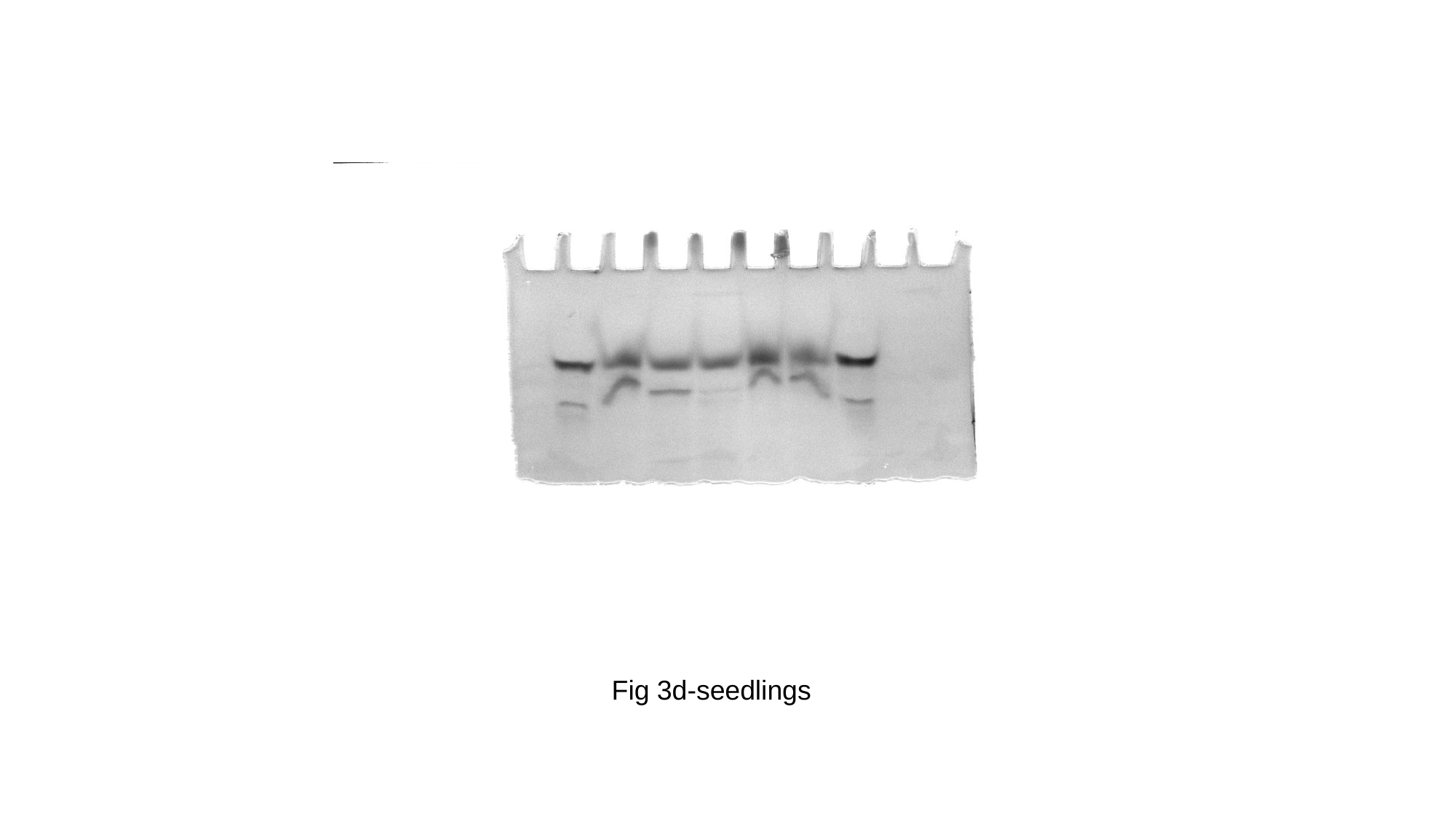

Fig 3d-seedlings

## Slide 7
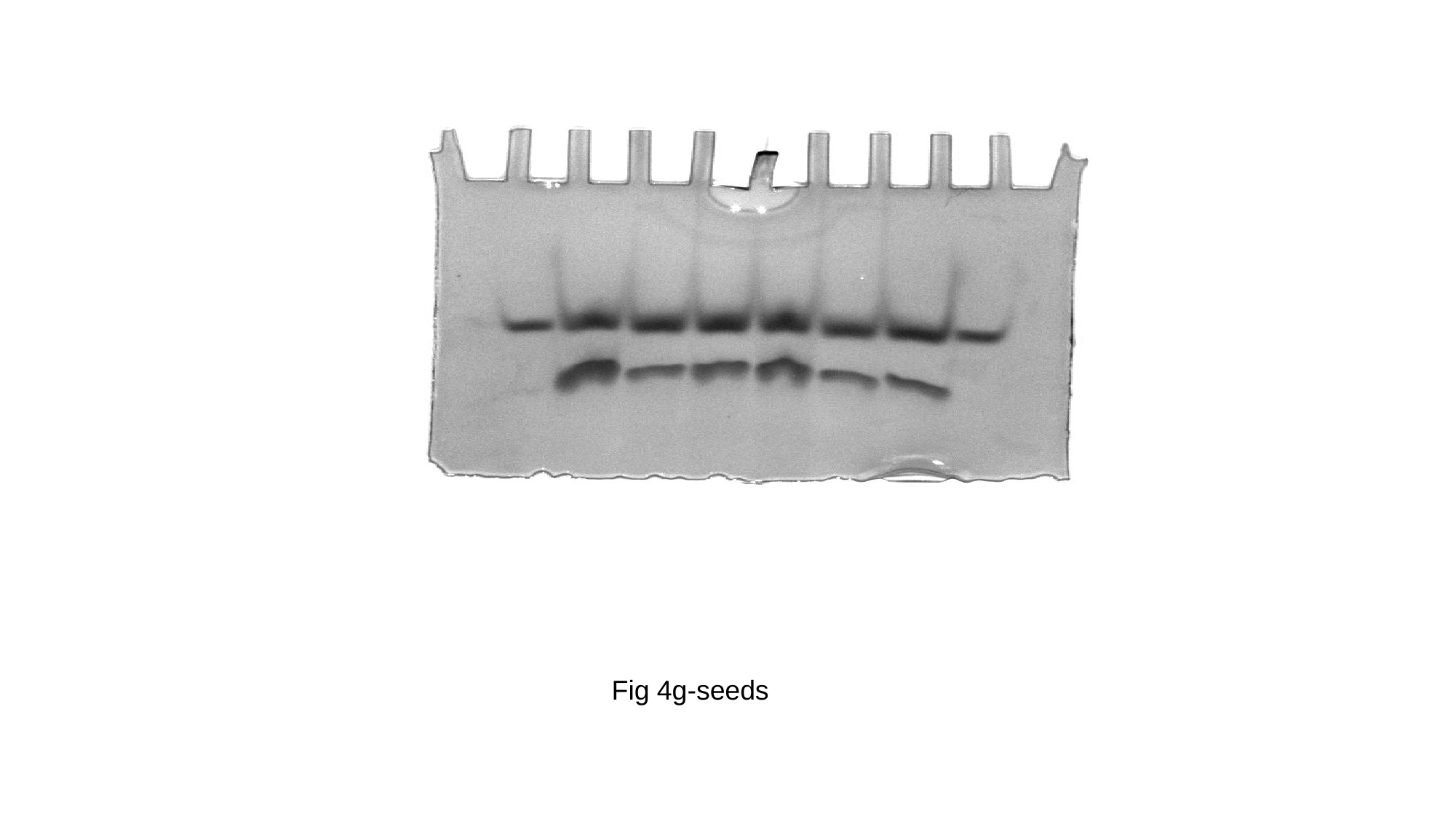

Fig 4g-seeds

## Slide 8
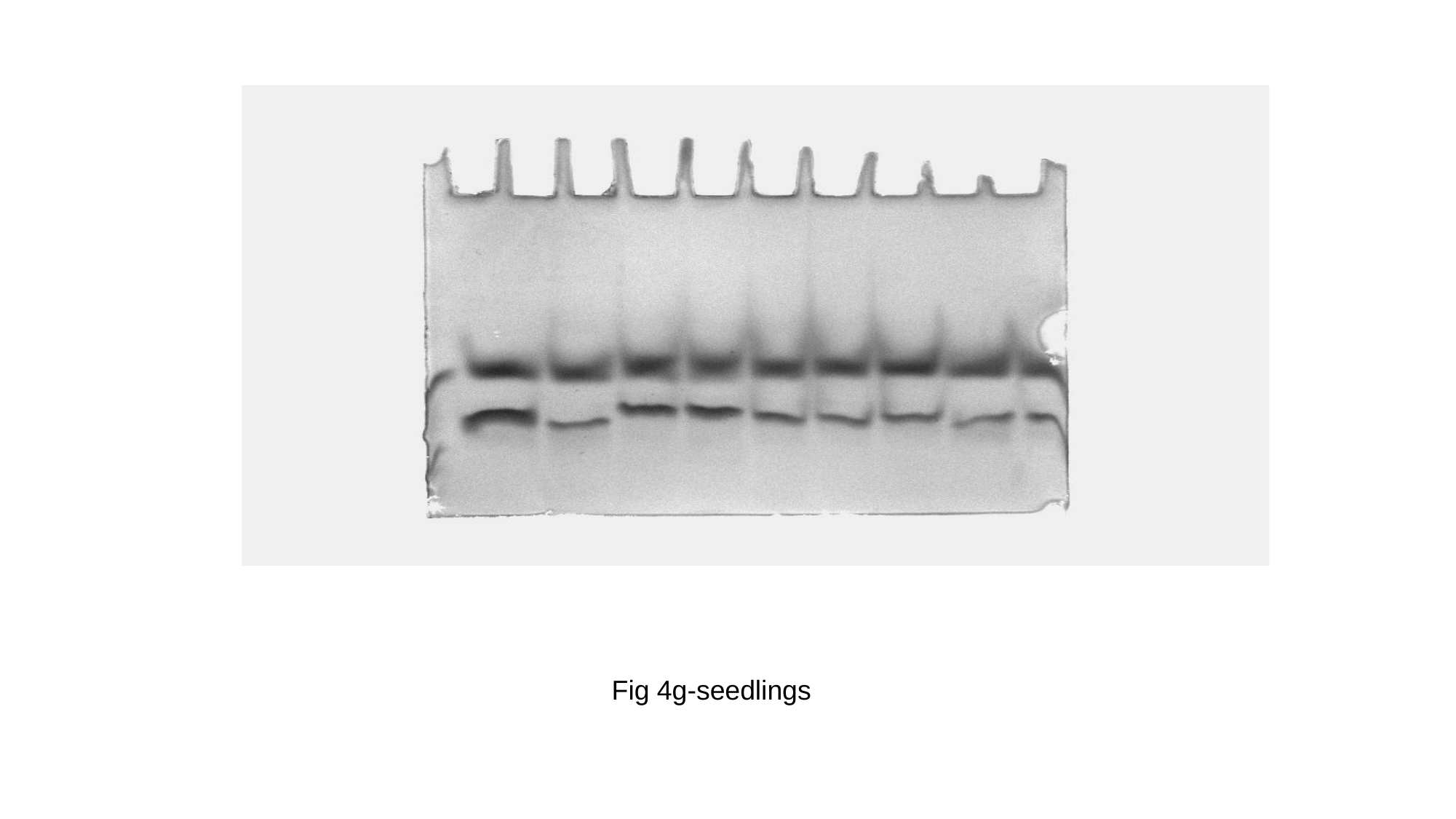

Fig 4g-seedlings

## Slide 9
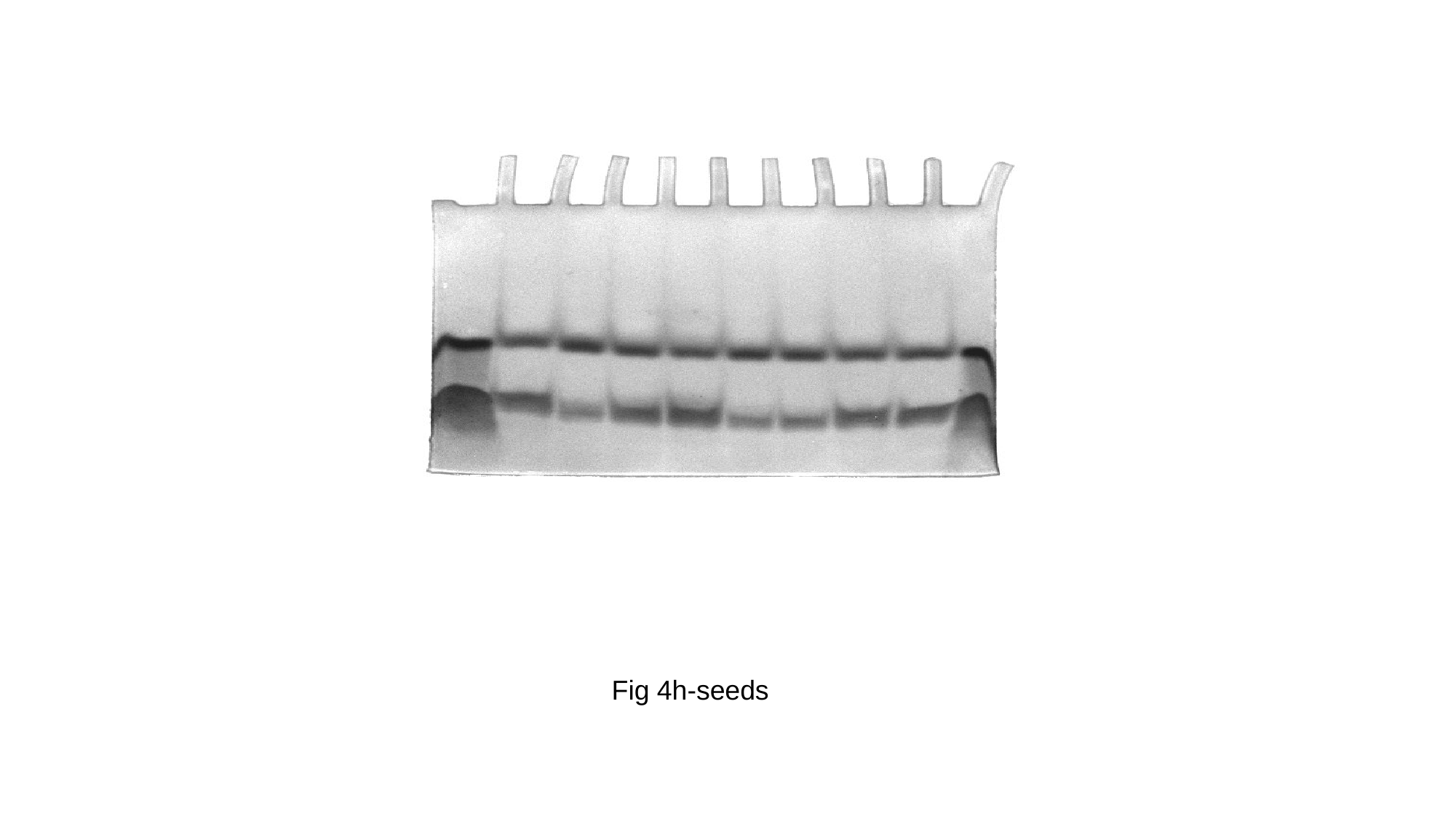

Fig 4h-seeds

## Slide 10
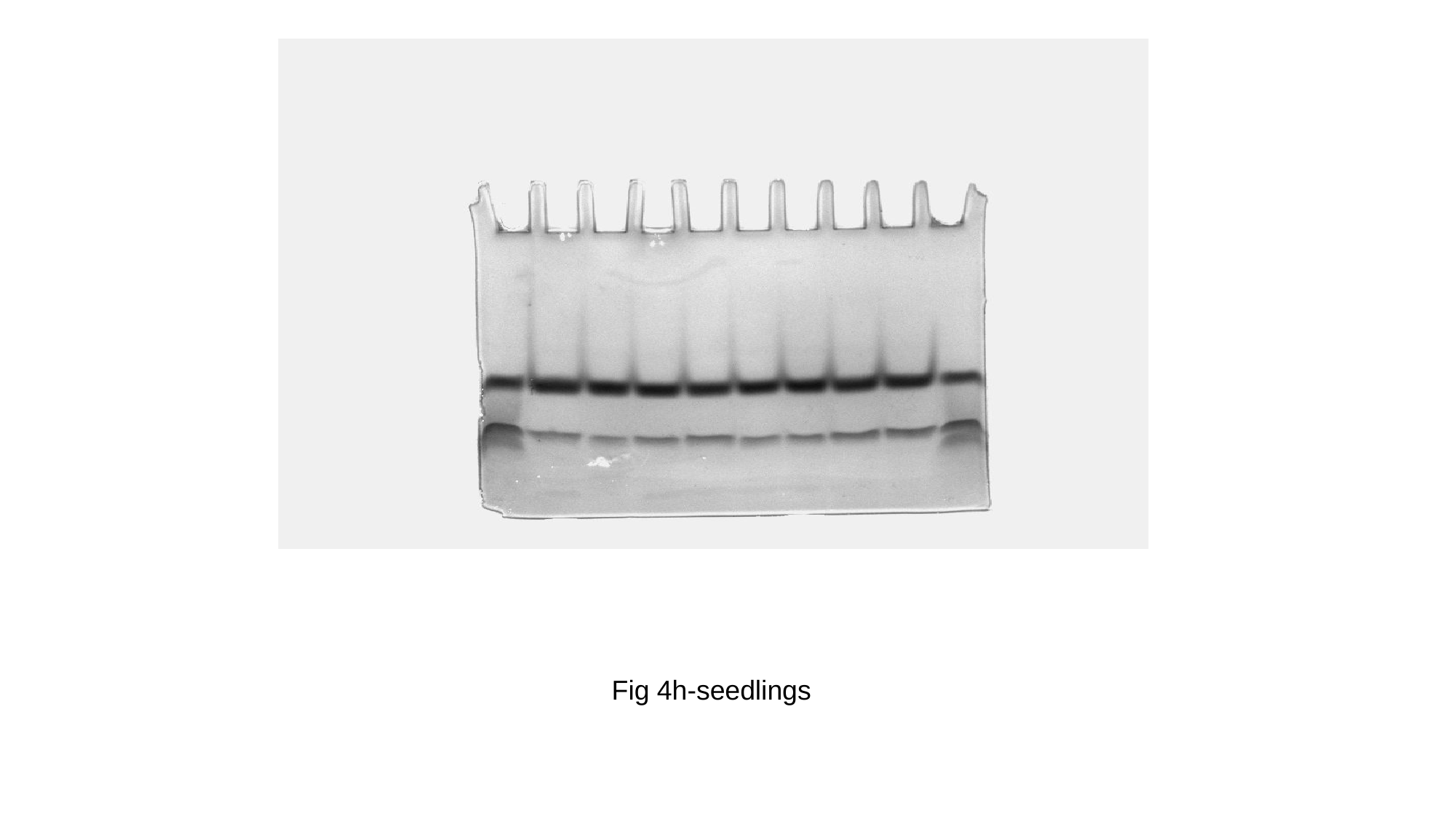

Fig 4h-seedlings

## Slide 11
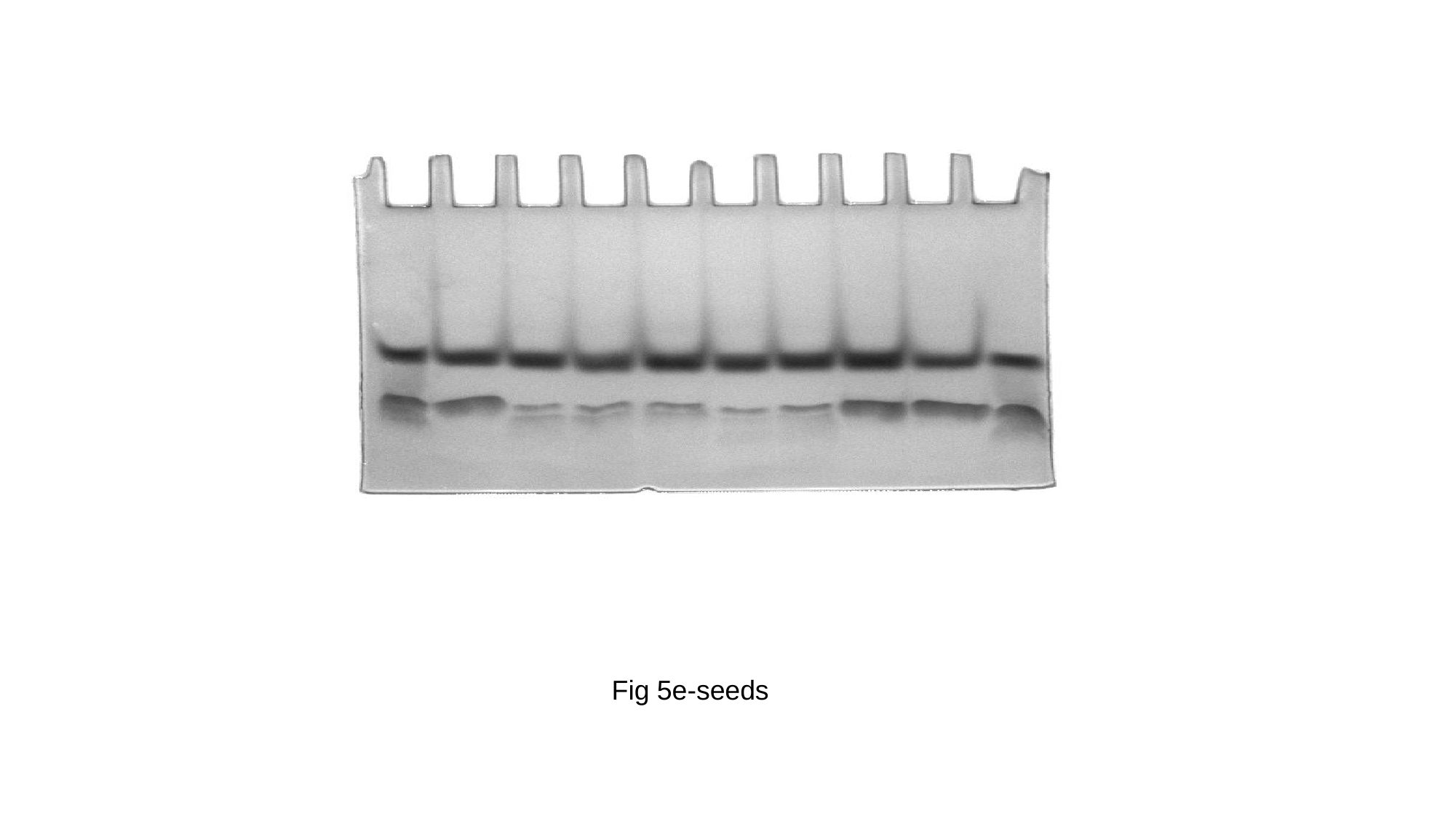

Fig 5e-seeds

## Slide 12
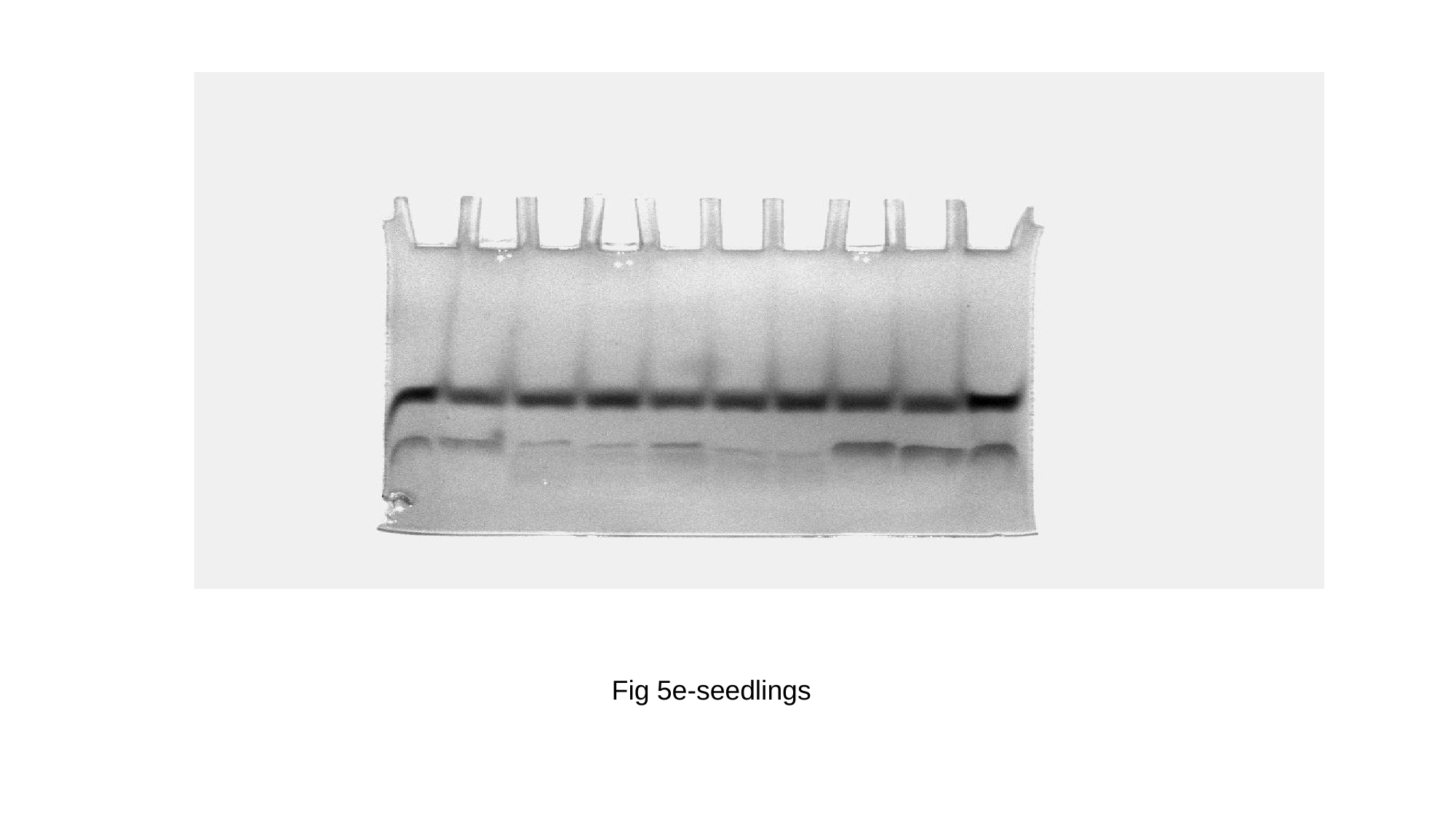

Fig 5e-seedlings

## Slide 13
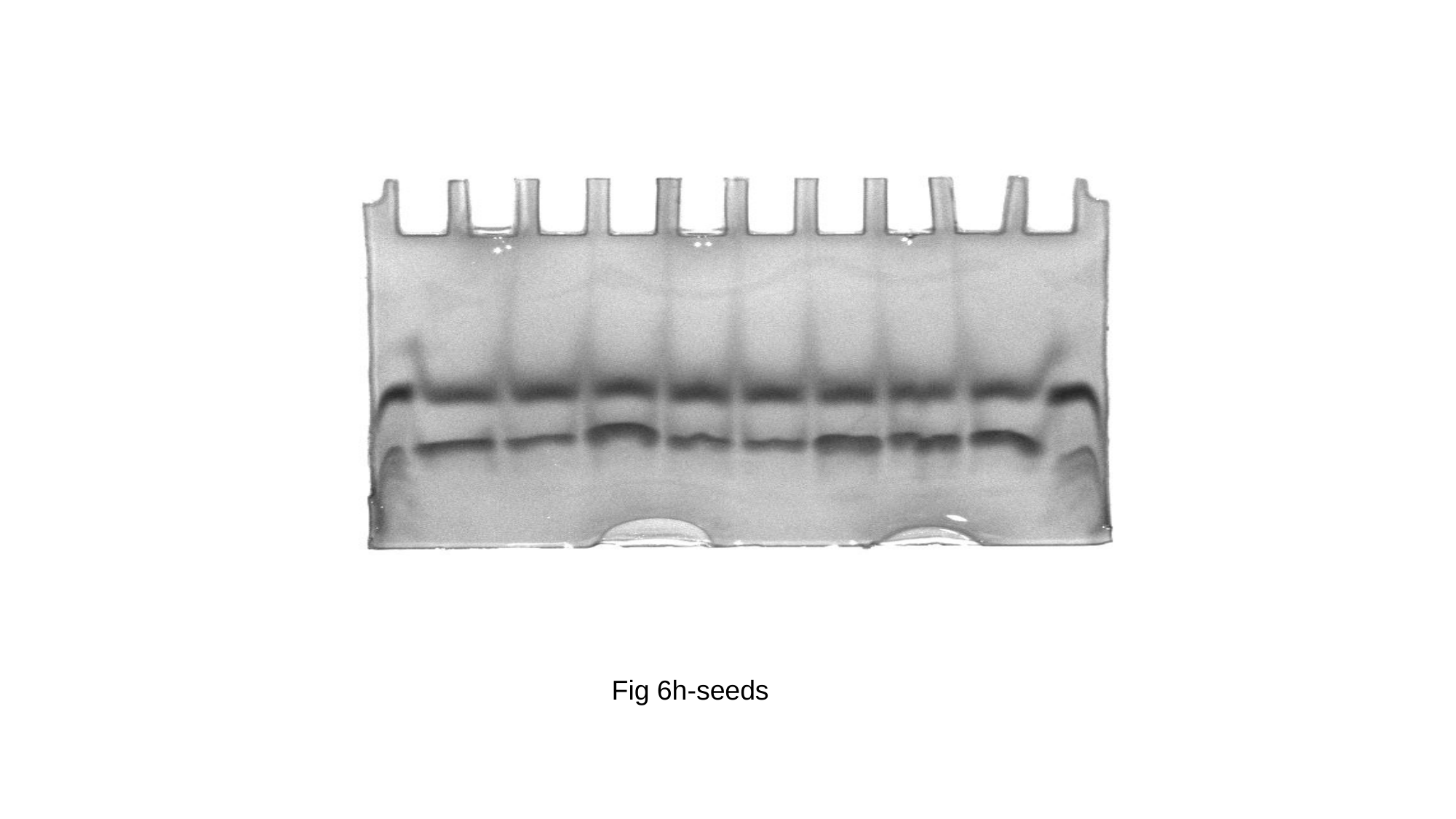

Fig 6h-seeds

## Slide 14
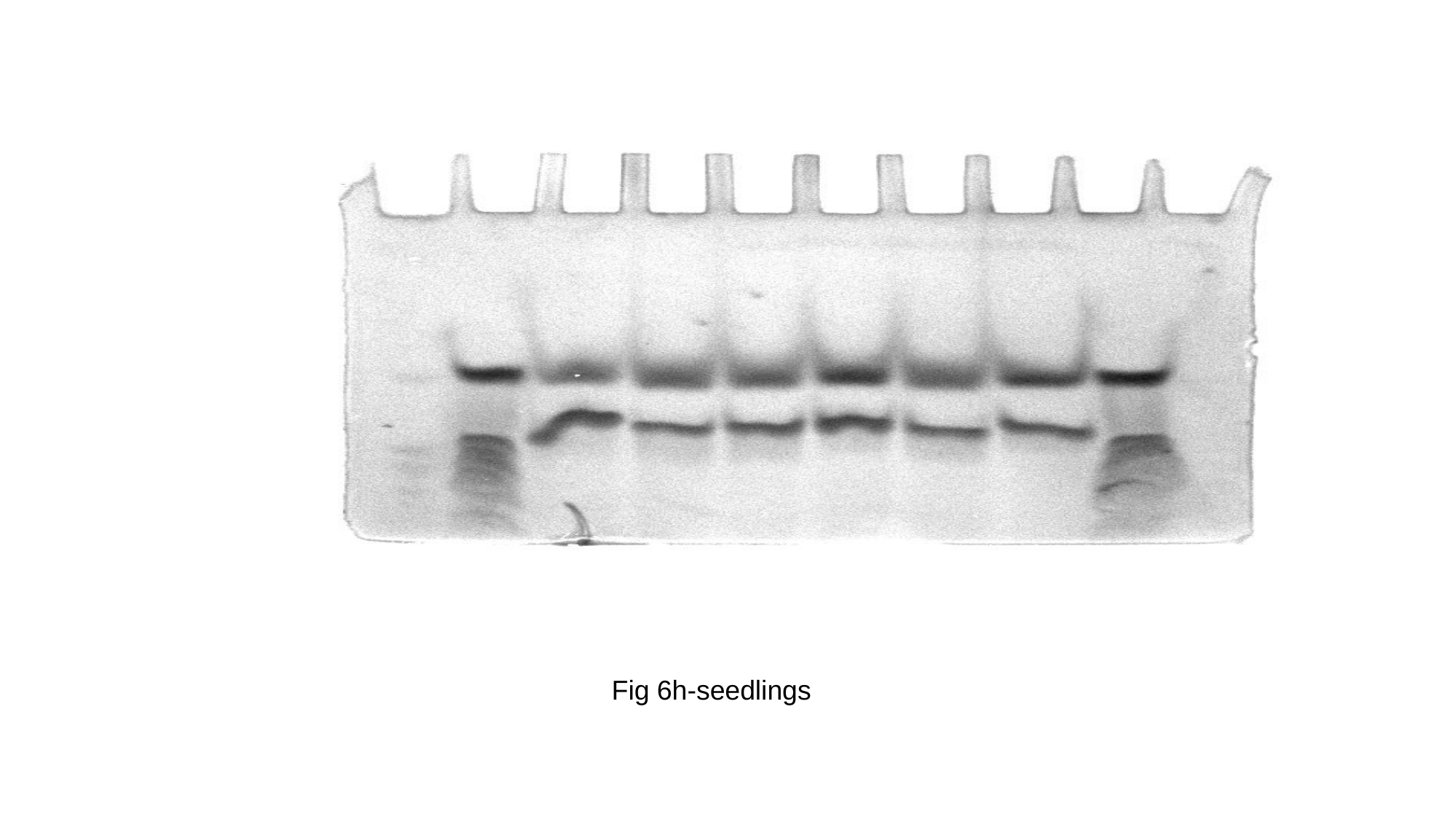

Fig 6h-seedlings

## Slide 15
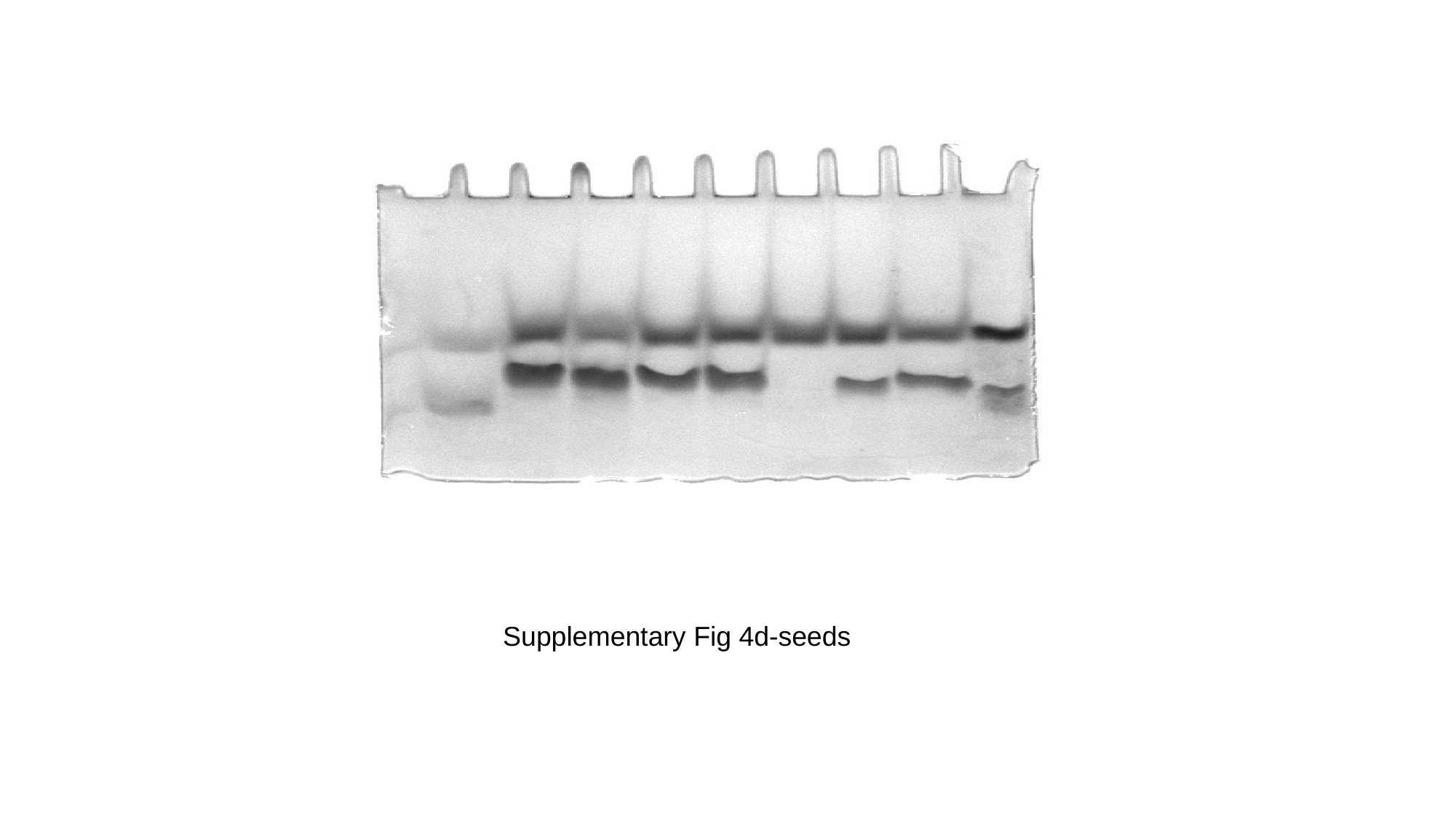

Supplementary Fig 4d-seeds

## Slide 16
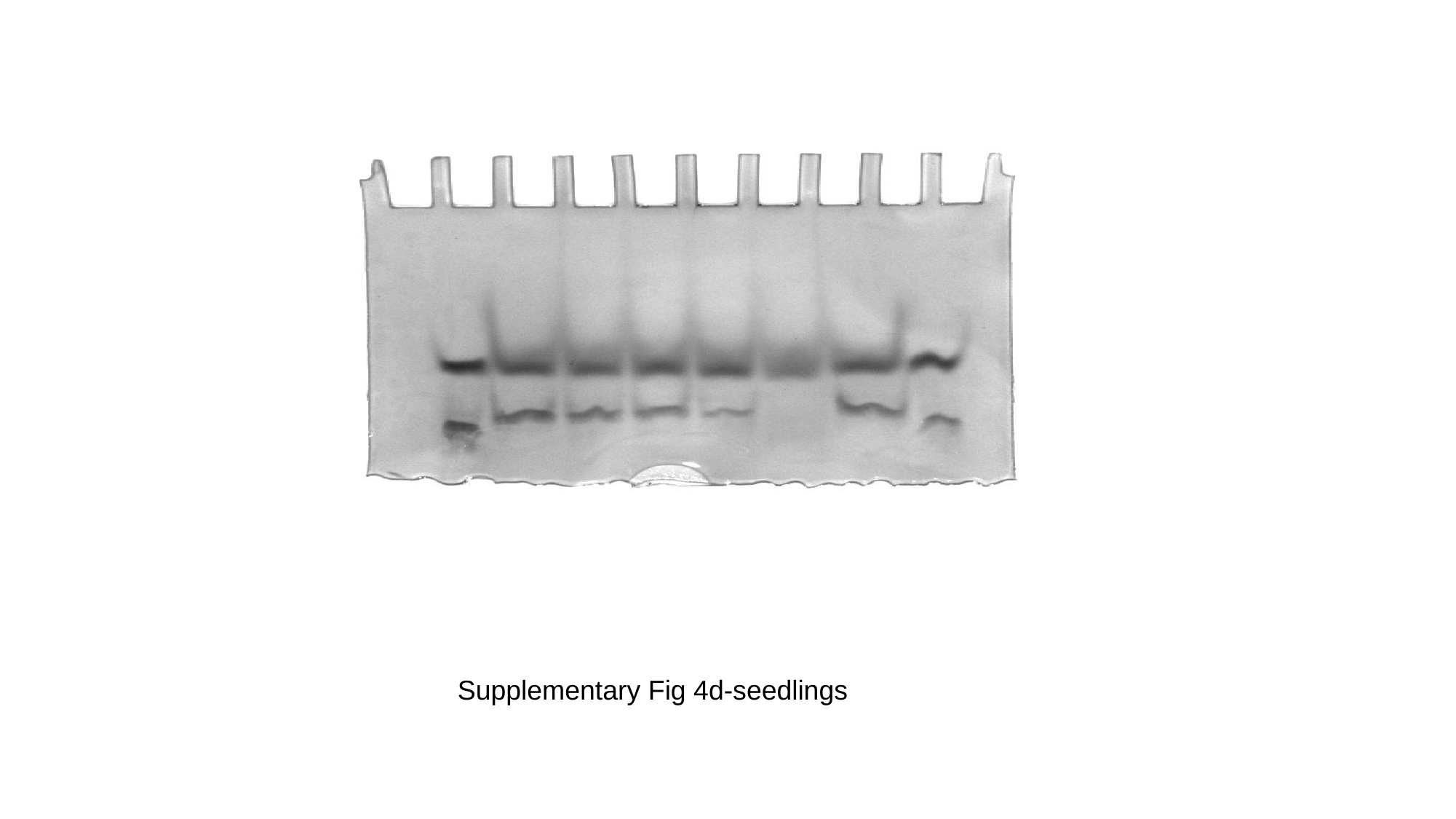

Supplementary Fig 4d-seedlings

## Slide 17
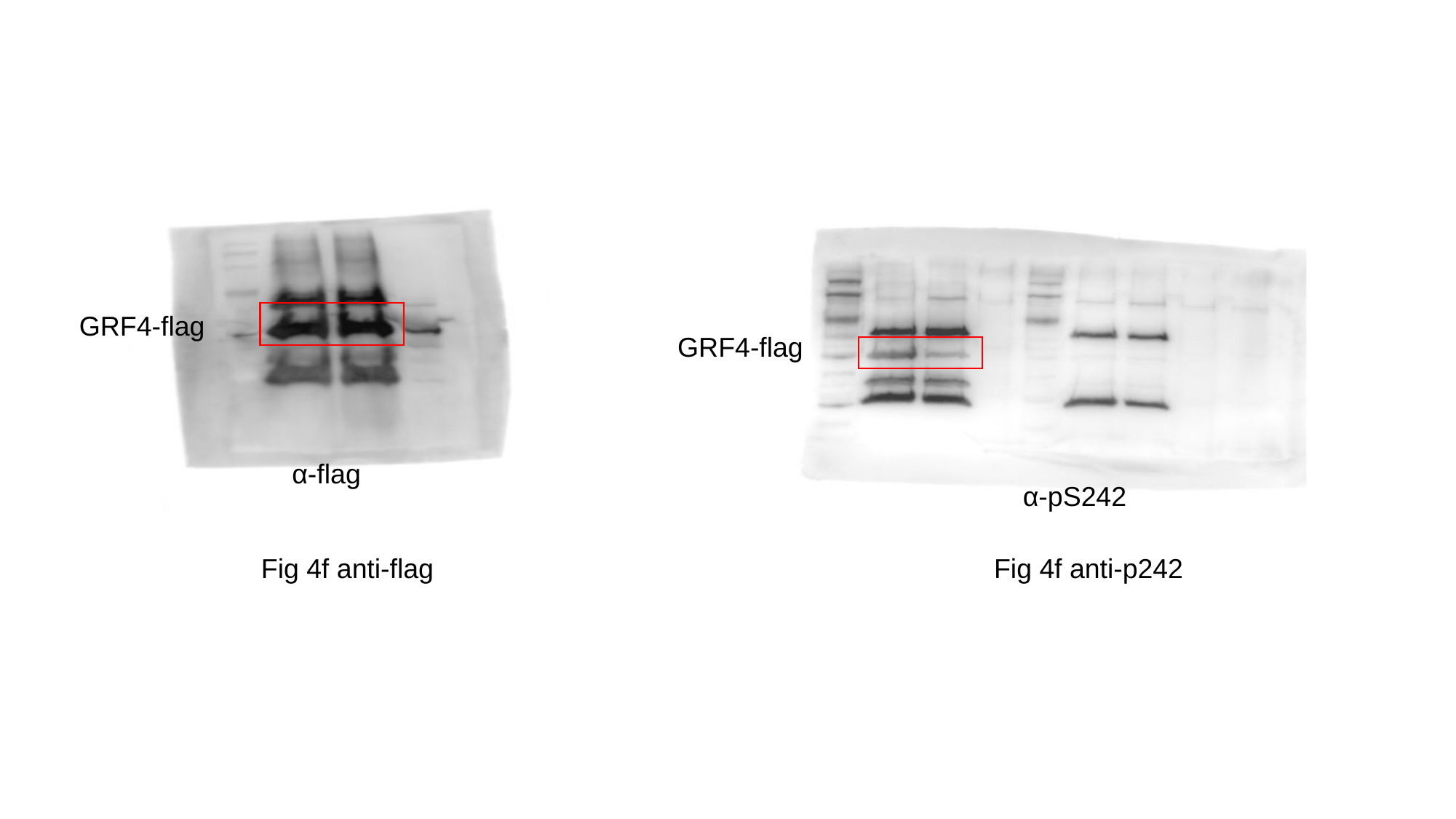

GRF4-flag
GRF4-flag
α-flag
α-pS242
 Fig 4f anti-flag
 Fig 4f anti-p242

## Slide 18
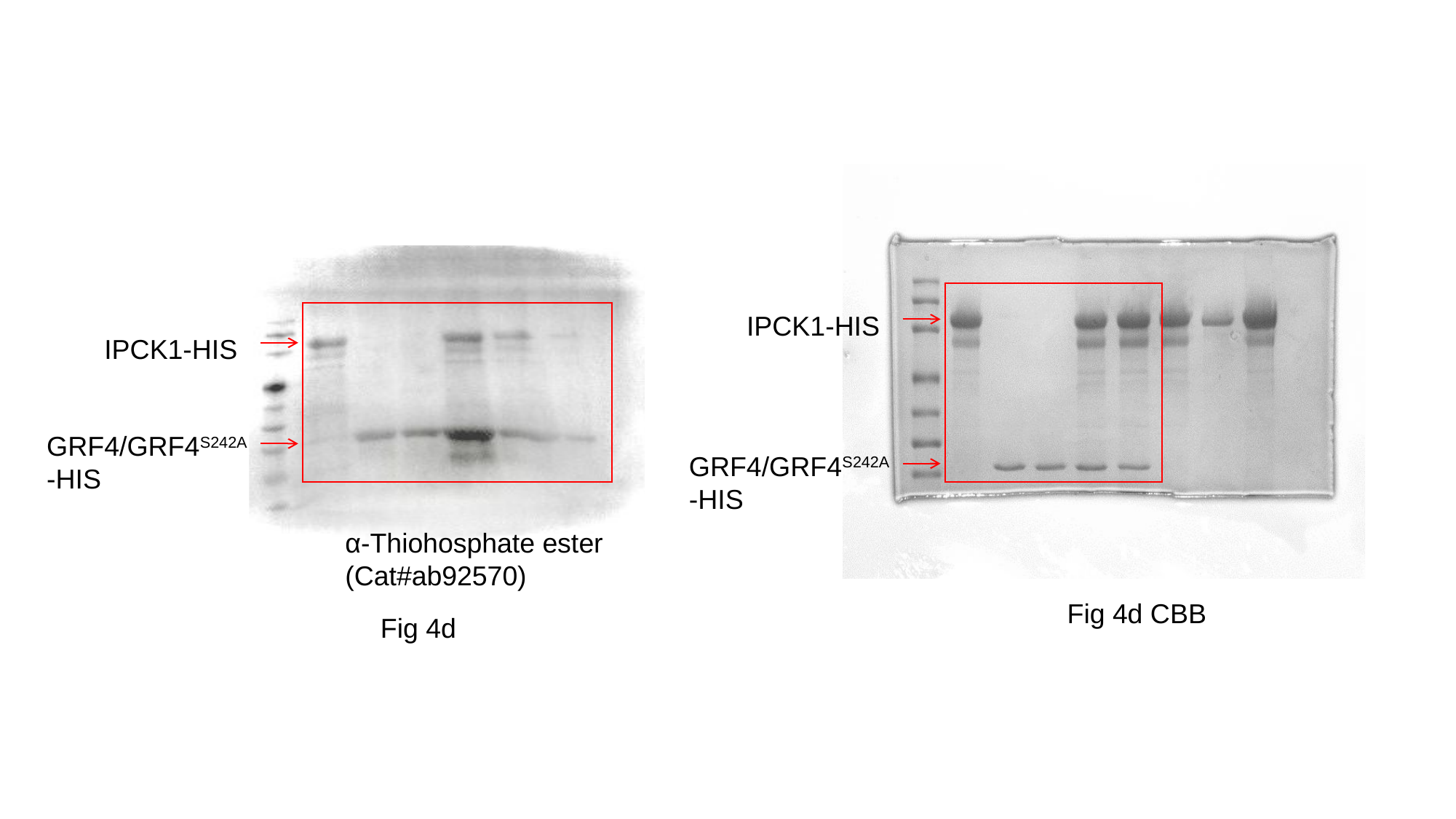

Fig 4d CBB
IPCK1-HIS
GRF4/GRF4S242A
-HIS
IPCK1-HIS
GRF4/GRF4S242A
-HIS
 Fig 4d
α-Thiohosphate ester (Cat#ab92570)

## Slide 19
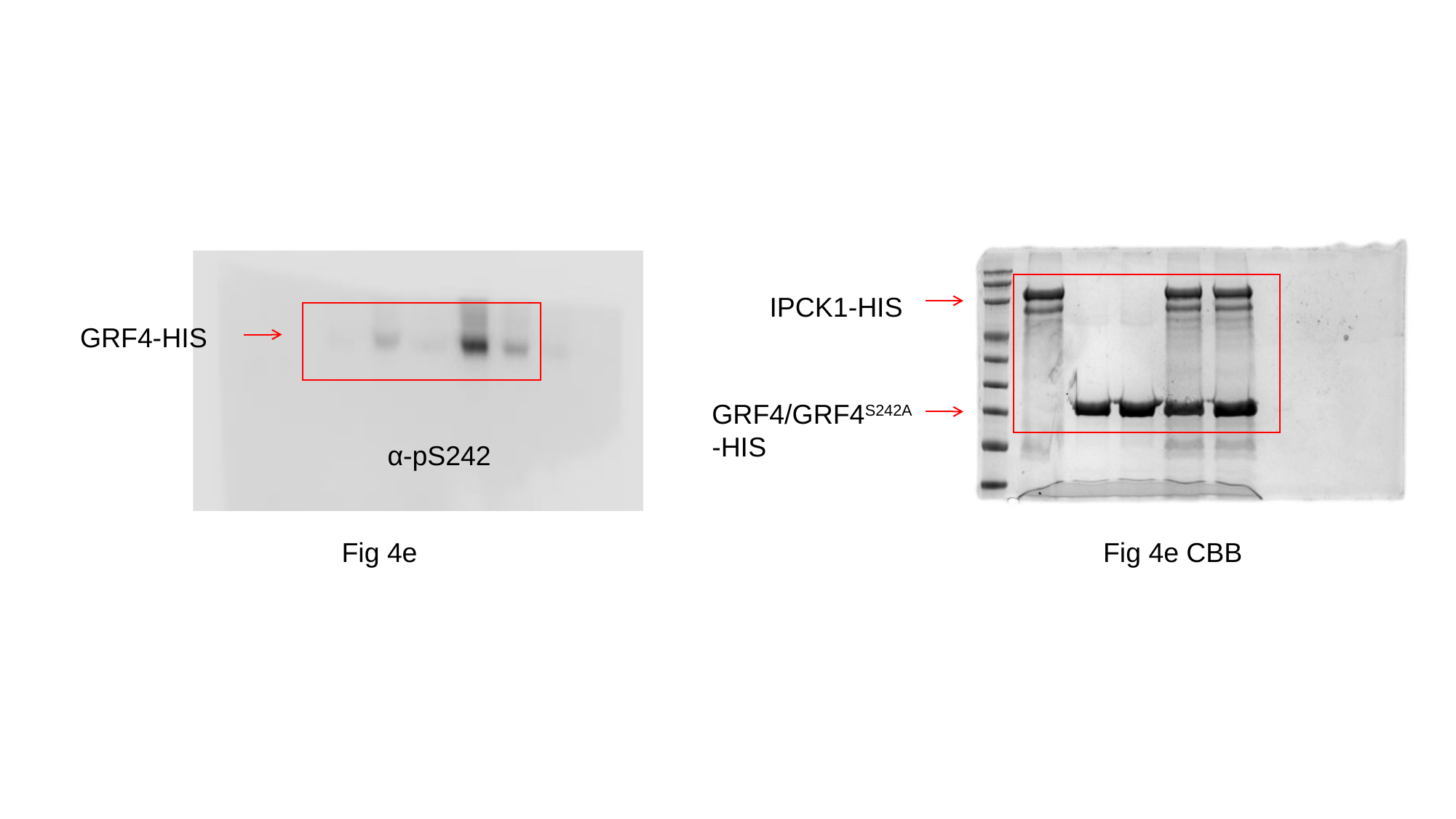

IPCK1-HIS
GRF4-HIS
GRF4/GRF4S242A
-HIS
α-pS242
 Fig 4e
 Fig 4e CBB

## Slide 20
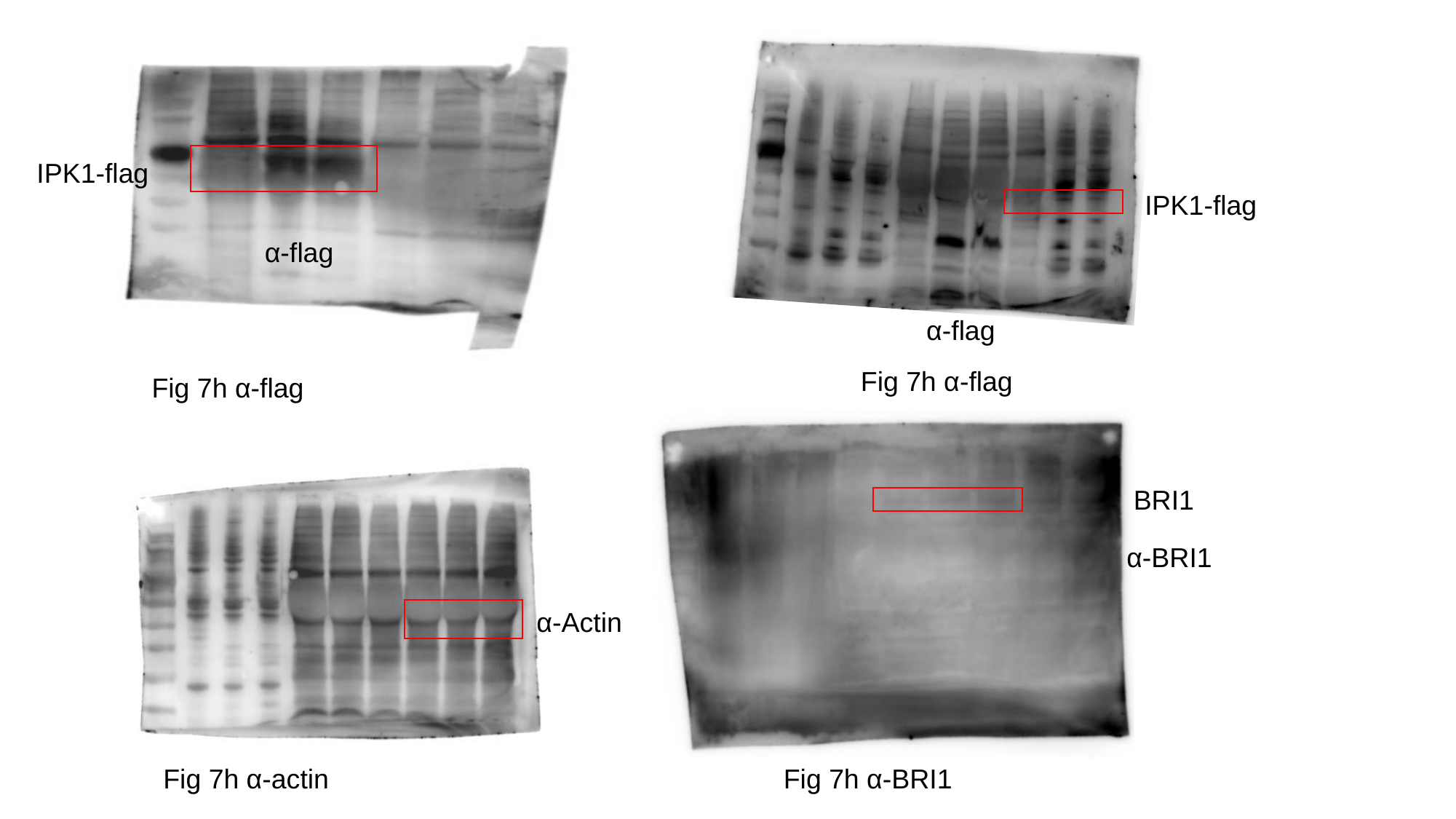

IPK1-flag
IPK1-flag
α-flag
α-flag
Fig 7h α-flag
Fig 7h α-flag
BRI1
α-BRI1
α-Actin
Fig 7h α-actin
Fig 7h α-BRI1

## Slide 21
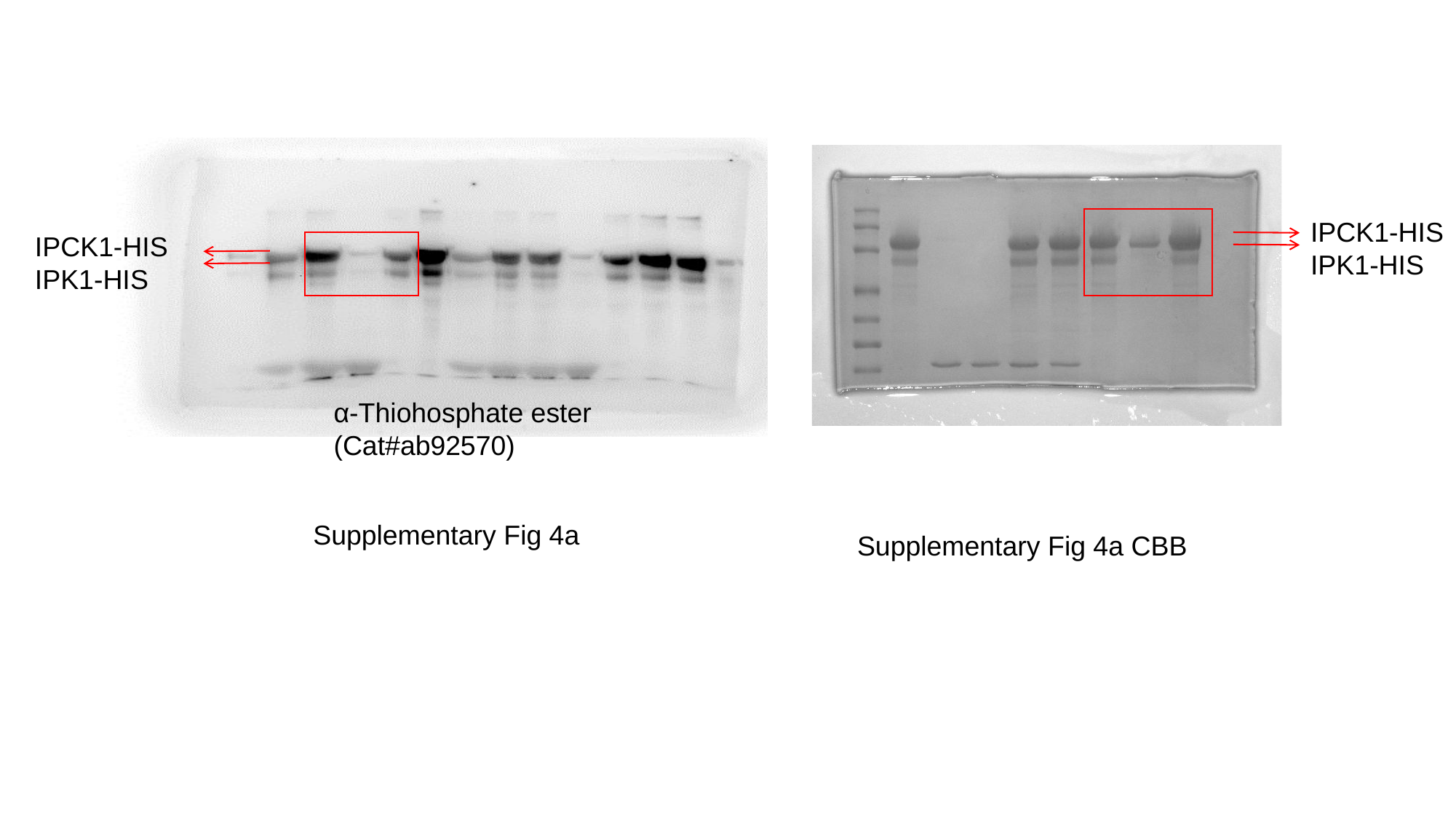

IPCK1-HIS
IPK1-HIS
IPCK1-HIS
IPK1-HIS
α-Thiohosphate ester (Cat#ab92570)
 Supplementary Fig 4a
 Supplementary Fig 4a CBB

## Slide 22
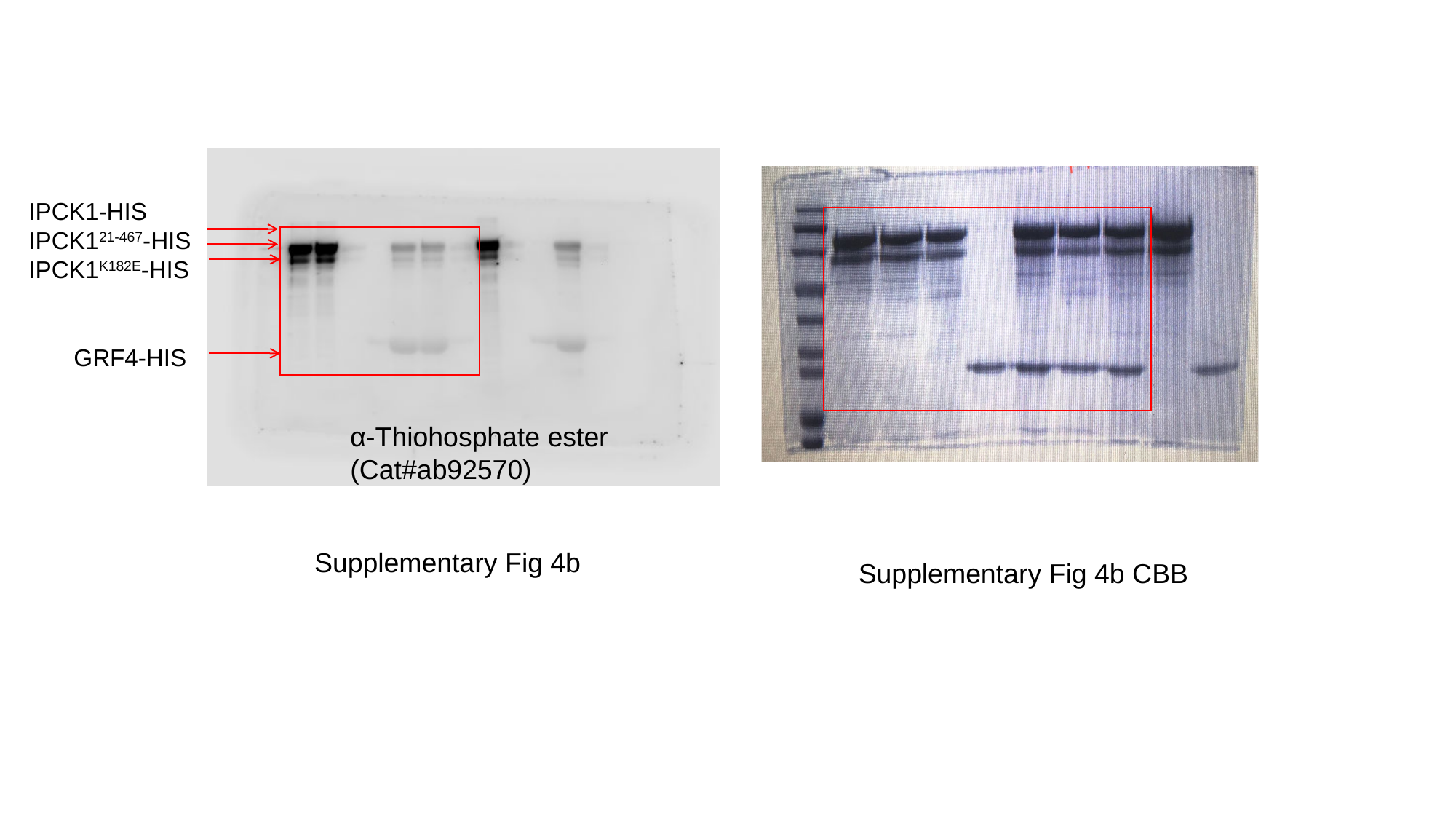

IPCK1-HIS
IPCK121-467-HIS
IPCK1K182E-HIS
GRF4-HIS
α-Thiohosphate ester (Cat#ab92570)
 Supplementary Fig 4b
 Supplementary Fig 4b CBB

## Slide 23
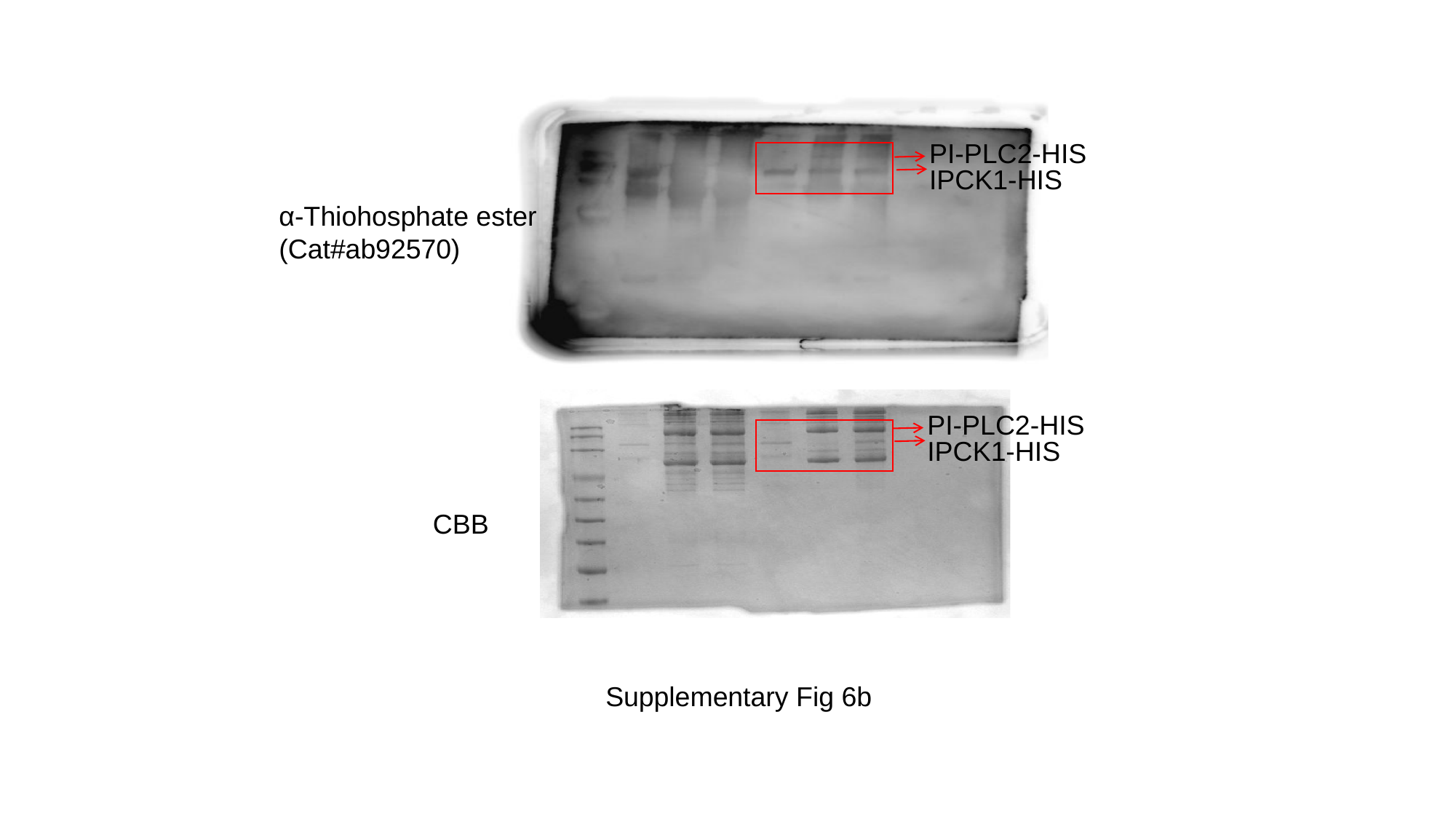

PI-PLC2-HIS
IPCK1-HIS
α-Thiohosphate ester (Cat#ab92570)
PI-PLC2-HIS
IPCK1-HIS
CBB
 Supplementary Fig 6b

## Slide 24
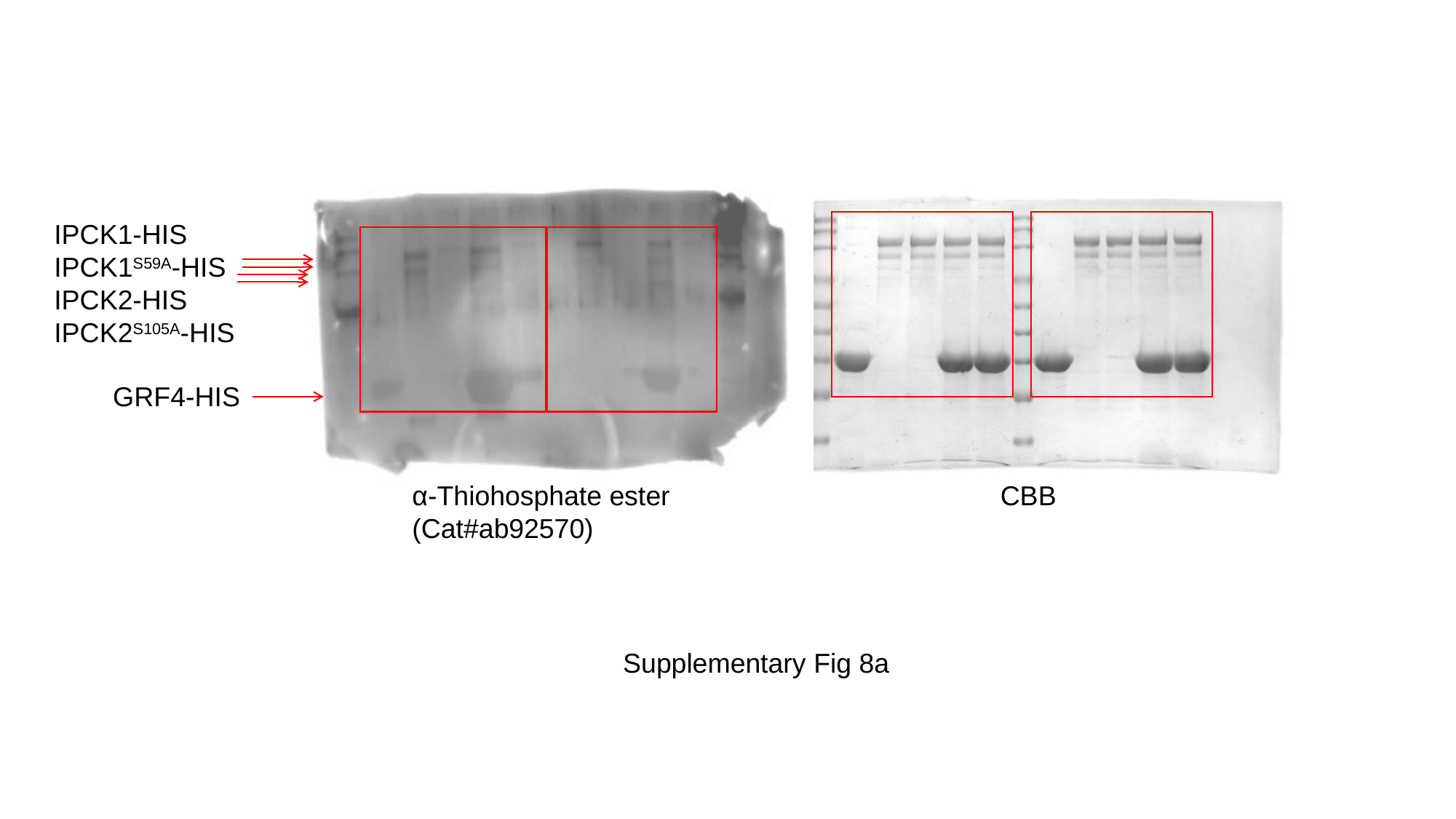

CBB
IPCK1-HIS
IPCK1S59A-HIS
IPCK2-HIS
IPCK2S105A-HIS
GRF4-HIS
α-Thiohosphate ester (Cat#ab92570)
 Supplementary Fig 8a
